# Changes in vaccine attitudes and recommendations among US Healthcare Personnel during the COVID-19 pandemic

**DOI:** 10.1038/s41541-024-00826-y

**Published:** 2024-02-28

**Authors:** Matthew Z. Dudley, Holly B. Schuh, Amanda Forr, Jana Shaw, Daniel A. Salmon

**Affiliations:** 1 Institute for Vaccine Safety, Johns Hopkins Bloomberg School of Public Health, Baltimore, MD USA; 2 Department of International Health, Johns Hopkins Bloomberg School of Public Health, Baltimore, MD USA; 3 Department of Epidemiology, Johns Hopkins Bloomberg School of Public Health, Baltimore, MD USA; 4 Element A LLC, Hershey, PA USA; 5 Division of Infectious Diseases, Department of Pediatrics, SUNY Upstate Medical University, Syracuse, NY USA; 6 Department of Health, Behavior and Society, Johns Hopkins Bloomberg School of Public Health, Baltimore, MD USA

**Keywords:** Health care, Vaccines

## Abstract

A recommendation from healthcare personnel (HCP) is a strong predictor of vaccination. This study aimed to measure how HCP vaccine attitudes and recommendations changed during the COVID-19 pandemic. HCP were surveyed in January 2023 using a double opt-in network panel. Survey responses were summarized and stratified by HCP type and COVID-19 booster status. Multivariable logistic regression models were fitted. Comparisons were made to a September 2021 survey, with differences tested for significance (*p* < 0.05) using Pearson’s *χ*^2^ Test. Nearly 82% of the 1207 HCP surveyed had received a COVID-19 booster, most commonly pediatricians (94%), followed by family medicine doctors (87%), pharmacists (74%), and nurses (73%) (*p* < 0.01). HCP with high trust in the Centers for Disease Control and Prevention (CDC) had nearly 6 times the odds (OR: 5.5; 95%CI: 3.9–7.7) of being boosted compared to HCP with low trust. From September 2021 to January 2023, the proportion of HCP recommending vaccines (COVID-19 and routine) to their patients decreased substantially for nearly all vaccines and patient populations specified. Trust in CDC also decreased (from 79 to 73%, *p* < 0.01), as did support for HCP COVID-19 vaccine mandates (from 65 to 46%, *p* < 0.01). HCP interest in additional online resources to improve their vaccine discussions with patients increased from 46 to 66% (*p* < 0.01). Additional regularly updated online resources from trusted medical sources that clarify progressing science and address dynamic public concerns are needed to improve vaccine confidence among HCP and help them support their patients’ decision-making.

## Introduction

The frequent updates to public health recommendations throughout the Coronavirus Disease 2019 (COVID-19) pandemic have caused public fatigue and confusion, and facilitated the unfortunate political polarization of vaccines in the United States (US)^1,2^. These factors have decreased public confidence in not only COVID-19 vaccines but also routine vaccines, threatening disease control efforts^3^. High coverage with updated vaccines is critical to ongoing efforts to minimize morbidity and mortality from COVID-19. Per a September 2022 national panel survey, only 63% of US adults were up-to-date on COVID-19 vaccines (e.g., having received a primary series and booster dose)^4^. However, nearly half (46%) of the adults not yet up-to-date were undecided about receiving further COVID-19 vaccines, signaling an opportunity to support their decision-making. Healthcare personnel (HCP) are the most frequently used and credible source of vaccine information for the public^5^, and a recommendation to vaccinate from a trusted HCP strongly predicts acceptance of COVID-19^6,7^ and other vaccines^8,9^, making HCP a critical source of vaccine information and decision-making support for their patients.

We previously conducted a cross-sectional survey of HCP in September 2021^10^, in which 94% of HCP had received (or intended to soon receive) a COVID-19 vaccine. Pediatricians had the highest vaccine coverage (98%), while family medicine doctors (96%) and pharmacists (94%) were close behind. In contrast, only 88% of physician assistants (PAs), nurse practitioners (NPs), and nurses were vaccinated against COVID-19. HCP not vaccinated against COVID-19 harbored similar vaccine concerns to the public; about half were concerned about side effects, and one-third were concerned with the speed of development. About three-quarters of HCP strongly recommended mRNA COVID-19 vaccines to their patients.

Herein we present our findings from a second cross-sectional survey of HCP conducted in January 2023, which was more than a year since the previous survey and since COVID-19 booster doses were first authorized^11^. We describe HCP COVID-19 booster status (and reasons for not vaccinating), as well as their trust in the US Centers for Disease Control and Prevention (CDC), the strength of recommendations to their patients for COVID-19 and other vaccines, the pandemic’s effect on their routine vaccination practices, and their vaccine resources and discussions with patients. We also examine differences in survey responses by HCP type, COVID-19 booster status, and urbanicity of HCP practice locations. Finally, we calculate changes in responses from the previous survey.

## Results

### Study population

Characteristics of the surveyed HCP (*n* = 1207) and their patient populations are shown in Table 1. Each of the four main HCP type categories (pediatricians, family medicine doctors, (PAs)/(NPs)/nurses, and pharmacists) made up about one quarter of the total sample. Nearly 89% of HCP regularly cared for COVID-19 patients; family medicine doctors cared for COVID-19 patients most frequently (93%), followed by PAs/NPs/nurses (89%), pediatricians (88%), then pharmacists (85%) (*p* = 0.02). Half of HCP (49%) practiced in suburban locations; 37% practiced in urban locations and 14% practiced in rural locations. Most HCP (96%) saw at least 10 patients per day. Most practices (97%) administered vaccines at the time of the survey, including the previous season’s (2020–2021) influenza vaccine (94%).

**Table 1:** Characteristics of participating healthcare personnel, their practices, and their patient populations

	Total	Pediatrician	Family medicine	PA, NP, Nurse	Pharmacist	*p*-value^a^
	*N* = 1207 (%)	*N* = 300 (%)	*N* = 300 (%)	*N* = 307 (%)	*N* = 300 (%)	
Practice characteristics						
Practice location, urban/suburban/rural						**<0.01**
Urban	446 (37.0)	116 (38.7)	91 (30.3)	122 (39.7)	117 (39.0)	
Suburban	590 (48.9)	164 (54.7)	157 (52.3)	132 (43.0)	137 (45.7)	
Rural	171 (14.2)	20 (6.7)	52 (17.3)	53 (17.3)	46 (15.3)	
U.S. region, assigned						**0.04**
Northeast	278 (23.0)	84 (28.0)	57 (19.0)	75 (24.4)	62 (20.7)	
Midwest	325 (26.9)	76 (25.3)	89 (29.7)	80 (26.1)	80 (26.7)	
South	402 (33.3)	92 (30.7)	89 (29.7)	107 (34.9)	114 (38.0)	
West	202 (16.7)	48 (16.0)	65 (21.7)	45 (14.7)	44 (14.7)	
Practice setting						**<0.01**
Private, independent practice	498 (41.3)	175 (58.3)	195 (65.0)	128 (41.7)	0 (0.0)	
Practice network/HMO	105 (8.7)	31 (10.3)	38 (12.7)	36 (11.7)	0 (0.0)	
Hospital or medical center	227 (18.8)	74 (24.7)	40 (13.3)	113 (36.8)	0 (0.0)	
Community health center/Federally Qualified Health Center (FQHC)	59 (4.9)	17 (5.7)	19 (6.3)	23 (7.5)	0 (0.0)	
Other	18 (1.5)	3 (1.0)	8 (2.7)	7 (2.3)	0 (0.0)	
Missing	300 (24.9)	0 (0.0)	0 (0.0)	0 (0.0)	300 (100.0)	
Average number of patients per day						**<0.01**
<10	44 (3.6)	3 (1.0)	10 (3.3)	18 (5.9)	13 (4.3)	
10–24	613 (50.8)	191 (63.7)	184 (61.3)	193 (62.9)	45 (15.0)	
≥25	550 (45.6)	106 (35.3)	106 (35.3)	96 (31.3)	242 (80.7)	
Service population						**<0.01**
Children (<18 yrs)	193 (16.0)	177 (59.0)	0 (0.0)	15 (4.9)	1 (0.3)	
Adults (≥18 yrs)	199 (16.5)	2 (0.7)	49 (16.3)	103 (33.6)	45 (15.0)	
Both children and adults	815 (67.5)	121 (40.3)	251 (83.7)	189 (61.6)	254 (84.7)	
Practice currently administers vaccines	1168 (96.8)	297 (99.0)	295 (98.3)	289 (94.1)	287 (95.7)	**<0.01**
Practice provided seasonal influenza vaccination: 2019–2020	597 (92.1)	140 (96.6)	124 (90.5)	133 (89.9)	200 (91.7)	0.14
Practice provided seasonal influenza vaccination: 2020–2021	609 (94.0)	143 (98.6)	128 (93.4)	130 (87.8)	208 (95.4)	**<0.01**
Practice provided seasonal influenza vaccination: 2021–2022	607 (93.7)	141 (97.2)	126 (92.0)	140 (94.6)	200 (91.7)	0.15
Practice provides COVID-19 vaccines	881 (73.0)	217 (72.3)	171 (57.0)	222 (72.3)	271 (90.3)	**<0.01**
Pfizer COVID-19 vax	758 (86.0)	201 (92.6)	138 (80.7)	196 (88.3)	223 (82.3)	**<0.01**
Moderna COVID-19 vax	632 (71.7)	116 (53.5)	122 (71.3)	173 (77.9)	221 (81.5)	**<0.01**
J&J COVID-19 vax	107 (12.1)	9 (4.1)	19 (11.1)	42 (18.9)	37 (13.7)	**<0.01**
Novavax COVID-19 vax	60 (6.8)	5 (2.3)	14 (8.2)	20 (9.0)	21 (7.7)	**0.02**
Strategies used to improve COVID-19 vaccine series completion:						
Paper-based reminder card	409 (46.4)	74 (34.1)	62 (36.3)	118 (53.2)	155 (57.2)	**<0.01**
Reminder telephone calls	347 (39.4)	82 (37.8)	53 (31.0)	93 (41.9)	119 (43.9)	**0.04**
Reminder text messages	299 (33.9)	60 (27.6)	36 (21.1)	81 (36.5)	122 (45.0)	**<0.01**
Reminder emails	283 (32.1)	56 (25.8)	54 (31.6)	74 (33.3)	99 (36.5)	0.09
Flagging patient charts	274 (31.1)	64 (29.5)	77 (45.0)	77 (34.7)	56 (20.7)	**<0.01**
Scheduling next dose at current visit	605 (68.7)	172 (79.3)	101 (59.1)	171 (77.0)	161 (59.4)	**<0.01**
Computerized immunization database/registry	342 (38.8)	91 (41.9)	67 (39.2)	95 (42.8)	89 (32.8)	0.09
Practice participates in the Vaccines for Children (VFC) program	612 (52.4)	250 (84.2)	139 (47.1)	125 (43.3)	98 (34.1)	**<0.01**
Practice uses Electronic Health Records (EHR)	952 (78.9)	270 (90.0)	268 (89.3)	285 (92.8)	129 (43.0)	**<0.01**
Patient characteristics						
Percent insured, private insurance						**<0.01**
<25%	172 (14.3)	42 (14.0)	29 (9.7)	47 (15.3)	54 (18.0)	
25–50%	439 (36.4)	76 (25.3)	106 (35.3)	120 (39.1)	137 (45.7)	
51–75%	352 (29.2)	80 (26.7)	110 (36.7)	88 (28.7)	74 (24.7)	
>75%	229 (19.0)	100 (33.3)	53 (17.7)	46 (15.0)	30 (10.0)	
Unsure	15 (1.2)	2 (0.7)	2 (0.7)	6 (2.0)	5 (1.7)	
Percent insured, Medicaid/CHIP						**<0.01**
<25%	608 (50.4)	124 (41.3)	213 (71.0)	140 (45.6)	131 (43.7)	
25–50%	411 (34.1)	106 (35.3)	61 (20.3)	109 (35.5)	135 (45.0)	
51–75%	108 (8.9)	38 (12.7)	13 (4.3)	35 (11.4)	22 (7.3)	
>75%	44 (3.6)	28 (9.3)	4 (1.3)	8 (2.6)	4 (1.3)	
Unsure	36 (3.0)	4 (1.3)	9 (3.0)	15 (4.9)	8 (2.7)	
Percent insured, Medicare						**<0.01**
<25%	470 (38.9)	237 (79.0)	91 (30.3)	94 (30.6)	48 (16.0)	
25–50%	496 (41.1)	13 (4.3)	173 (57.7)	132 (43.0)	178 (59.3)	
51–75%	130 (10.8)	2 (0.7)	28 (9.3)	52 (16.9)	48 (16.0)	
>75%	44 (3.6)	0 (0.0)	6 (2.0)	18 (5.9)	20 (6.7)	
Unsure	67 (5.6)	48 (16.0)	2 (0.7)	11 (3.6)	6 (2.0)	
Percent insured, Uninsured						**<0.01**
<25%	1,058 (87.7)	273 (91.0)	274 (91.3)	254 (82.7)	257 (85.7)	
25–50%	51 (4.2)	5 (1.7)	5 (1.7)	19 (6.2)	22 (7.3)	
51–75%	7 (0.6)	0 (0.0)	0 (0.0)	5 (1.6)	2 (0.7)	
>75%	7 (0.6)	0 (0.0)	2 (0.7)	4 (1.3)	1 (0.3)	
Unsure	84 (7.0)	22 (7.3)	19 (6.3)	25 (8.1)	18 (6.0)	
Percent race/ethnicity, Hispanic/Latino						**0.02**
<25%	754 (62.5)	184 (61.3)	204 (68.0)	172 (56.0)	194 (64.7)	
25–50%	352 (29.2)	90 (30.0)	79 (26.3)	109 (35.5)	74 (24.7)	
51–75%	70 (5.8)	18 (6.0)	12 (4.0)	20 (6.5)	20 (6.7)	
>75%	21 (1.7)	7 (2.3)	5 (1.7)	3 (1.0)	6 (2.0)	
Unsure	10 (0.8)	1 (0.3)	0 (0.0)	3 (1.0)	6 (2.0)	
Percent race/ethnicity, Black/African American						**<0.01**
<25%	711 (58.9)	173 (57.7)	207 (69.0)	151 (49.2)	180 (60.0)	
25–50%	399 (33.1)	111 (37.0)	83 (27.7)	121 (39.4)	84 (28.0)	
51–75%	66 (5.5)	10 (3.3)	10 (3.3)	26 (8.5)	20 (6.7)	
>75%	21 (1.7)	5 (1.7)	0 (0.0)	5 (1.6)	11 (3.7)	
Unsure	10 (0.8)	1 (0.3)	0 (0.0)	4 (1.3)	5 (1.7)	
Percent race/ethnicity, Asian						**0.02**
<25%	1,035 (85.7)	255 (85.0)	261 (87.0)	260 (84.7)	259 (86.3)	
25–50%	142 (11.8)	40 (13.3)	35 (11.7)	36 (11.7)	31 (10.3)	
51–75%	12 (1.0)	2 (0.7)	3 (1.0)	7 (2.3)	0 (0.0)	
>75%	7 (0.6)	1 (0.3)	1 (0.3)	0 (0.0)	5 (1.7)	
Unsure	11 (0.9)	2 (0.7)	0 (0.0)	4 (1.3)	5 (1.7)	
Percent race/ethnicity, Other minority group						**0.03**
<25%	202 (16.7)	45 (15.0)	43 (14.3)	53 (17.3)	61 (20.3)	
25–50%	24 (2.0)	5 (1.7)	2 (0.7)	11 (3.6)	6 (2.0)	
51–75%	15 (1.2)	2 (0.7)	3 (1.0)	4 (1.3)	6 (2.0)	
>75%	9 (0.7)	0 (0.0)	2 (0.7)	7 (2.3)	0 (0.0)	
Unsure	117 (9.7)	27 (9.0)	12 (4.0)	42 (13.7)	36 (12.0)	
Missing	840 (69.6)	221 (73.7)	238 (79.3)	190 (61.9)	191 (63.7)	
Provider characteristics						
Current medical profession						**<0.01**
Physician	600 (49.7)	300 (100.0)	300 (100.0)	0 (0.0)	0 (0.0)	
Physician Assistant	100 (8.3)	0 (0.0)	0 (0.0)	100 (32.6)	0 (0.0)	
Nurse Practitioner	105 (8.7)	0 (0.0)	0 (0.0)	105 (34.2)	0 (0.0)	
Nurse (RN or LPN)	102 (8.5)	0 (0.0)	0 (0.0)	102 (33.2)	0 (0.0)	
Pharmacist	300 (24.9)	0 (0.0)	0 (0.0)	0 (0.0)	300 (100.0)	
Specialty						**<0.01**
Internal Medicine	118 (9.8)	0 (0.0)	0 (0.0)	118 (38.4)	0 (0.0)	
Family Practice	458 (37.9)	0 (0.0)	300 (100.0)	158 (51.5)	0 (0.0)	
General Pediatrics	331 (27.4)	300 (100.0)	0 (0.0)	31 (10.1)	0 (0.0)	
Missing	300 (24.9)	0 (0.0)	0 (0.0)	0 (0.0)	300 (100.0)	
Highest clinical degree						**<0.01**
Associate degree	22 (1.8)	0 (0.0)	0 (0.0)	22 (7.2)	0 (0.0)	
Bachelor’s degree	162 (13.4)	0 (0.0)	0 (0.0)	65 (21.2)	97 (32.3)	
Master’s degree	206 (17.1)	0 (0.0)	0 (0.0)	192 (62.5)	14 (4.7)	
Doctorate level	811 (67.2)	300 (100.0)	300 (100.0)	26 (8.5)	185 (61.7)	
Missing	6 (0.5)	0 (0.0)	0 (0.0)	2 (0.7)	4 (1.3)	
Graduation year, highest completed clinical degree						**<0.01**
<1980	52 (4.3)	19 (6.3)	12 (4.0)	5 (1.6)	16 (5.3)	
1980–1989	195 (16.2)	68 (22.7)	77 (25.7)	13 (4.2)	37 (12.3)	
1990–1999	318 (26.3)	89 (29.7)	110 (36.7)	41 (13.4)	78 (26.0)	
2000–2009	380 (31.5)	90 (30.0)	74 (24.7)	111 (36.2)	105 (35.0)	
2010–2021	247 (20.5)	33 (11.0)	27 (9.0)	125 (40.7)	62 (20.7)	
Missing	15 (1.2)	1 (0.3)	0 (0.0)	12 (3.9)	2 (0.7)	
Race/Ethnicity						**<0.01**
White	832 (68.9)	189 (63.0)	195 (65.0)	246 (80.1)	202 (67.3)	
Asian	158 (13.1)	49 (16.3)	57 (19.0)	8 (2.6)	44 (14.7)	
Black	48 (4.0)	12 (4.0)	9 (3.0)	20 (6.5)	7 (2.3)	
Hispanic	37 (3.1)	11 (3.7)	5 (1.7)	12 (3.9)	9 (3.0)	
Other	38 (3.1)	14 (4.7)	12 (4.0)	6 (2.0)	6 (2.0)	
Missing	94 (7.8)	25 (8.3)	22 (7.3)	15 (4.9)	32 (10.7)	
Regularly taken care of COVID-19 patients	1,069 (88.6)	265 (88.3)	278 (92.7)	272 (88.6)	254 (84.7)	**0.02**
Received at least one COVID-19 vaccine	1,154 (95.6)	299 (99.7)	291 (97.0)	282 (91.9)	282 (94.0)	**<0.01**
Received at least one COVID-19 booster dose	989 (81.9)	281 (93.7)	261 (87.0)	225 (73.3)	222 (74.0)	**<0.01**
COVID-19 vaccination should be _____ for healthcare workers						**<0.01**
Voluntary	464 (38.4)	68 (22.7)	106 (35.3)	156 (50.8)	134 (44.7)	
Mandated	560 (46.4)	183 (61.0)	151 (50.3)	112 (36.5)	114 (38.0)	
Not sure	183 (15.2)	49 (16.3)	43 (14.3)	39 (12.7)	52 (17.3)	
High Trust in CDC ^b^	883 (73.2)	244 (81.3)	214 (71.3)	216 (70.4)	209 (69.7)	**<0.01**

*PA* Physician Assistant, *NP* Nurse Practitioner; *CDC* Centers for Disease Control and Prevention.

^a^Boldface indicates statistical significance (*p* < 0.05) using Pearson’s *χ*^2^ test.

^b^See Supplementary Table 1.

### COVID-19 vaccination

Nearly all HCP (96%) had received at least one COVID-19 vaccine, and nearly 82% had received at least one booster dose as well (Table 1). Pediatricians were the most frequently boosted (94%), followed by family medicine doctors (87%), pharmacists (74%), and PAs/NPs/nurses (73%) (*p* < 0.01). Both PAs/NPs/nurses and pharmacists had one-fifth the odds (OR: 0.2; 95%CI: 0.1–0.3) of being boosted compared to pediatricians, and family medicine doctors had one-half the odds (OR: 0.5; 95%CI: 0.2–0.9) of being boosted compared to pediatricians (Table 2). The odds ratios (ORs) comparing pediatricians to PAs/NPs/nurses and pharmacists remained significant (*p* < 0.05) even after adjusting for trust in CDC and other relevant sociodemographic characteristics.

**Table 2:** Odds of being boosted against COVID-19 by type of healthcare personnel and trust in CDC

	Total	Not Boosted	Boosted	Boosted (versus not)				
Covariate	*N* = 1207	*N* = 218 (%)	*N* = 989 (%)	*p*-value^a^	OR	95% CI	aOR^b^	95% CI
HCP type				**<0.01**				
Pediatrician	300	19 (6.3)	281 (93.7)	–	Ref.	–	Ref.	–.
Family medicine	300	39 (13.0)	261 (87.0)	–	**0.5**	**(0.2, 0.9)**	0.5	(0.3, 1.1)
PA, NP, Nurse	307	82 (26.7)	225 (73.3)	–	**0.2**	**(0.1, 0.3)**	**0.2**	**(0.1, 0.4)**
Pharmacist	300	78 (26.0)	222 (74.0)	–	**0.2**	**(0.1, 0.3)**	**0.2**	**(0.1, 0.4)**
Trust in CDC^c^				**<0.01**				
Low	324	126 (38.9)	198 (61.1)	–	Ref.	–	Ref.	–
High	883	92 (10.4)	791 (89.6)	–	**5.5**	**(3.9, 7.7)**	**5.5**	**(3.7, 8.1)**

*OR* Odds Ratio, *aOR* adjusted Odds Ratio, *PA* Physician Assistant; *NP* Nurse Practitioner, *CDC* Centers for Disease Control and Prevention; *HCP* Healthcare Personnel.

^a^Using the Pearson chi-square test at significance level of alpha=5%; bold indicates statistical significance (*p* < 0.05).

^b^HCP type adjusted for trust in CDC and vice versa; both HCP type and trust in CDC adjusted for sociodemographic characteristics shown to be associated with COVID-19 booster status, namely graduation year (to approximate age), race/ethnicity, region, and practice location (urban/suburban/rural); bold indicates statistical significance (*p* < 0.05).

^c^See Supplementary Table 1.

Bold indicates statistical significance (*p*  <  0.05).

### Trust in CDC

Nearly three-quarters of HCP had high trust in CDC (73.2%), with high trust most common among pediatricians (81%), followed by family medicine doctors (71%), PAs/NPs/nurses (70%), and pharmacists (70%) (*p* < 0.01) (Table 1). Most HCP with high trust in CDC (90%) were boosted, compared to 61% of HCP with low trust in CDC (*p* < 0.01) (Table 2). HCP with high trust in CDC had nearly 6 times the odds (OR: 5.5; 95%CI: 3.9–7.7) of being boosted compared to HCP with low trust in CDC, an association that remained significant (*p* < 0.05) even after adjusting for HCP type and other relevant sociodemographic characteristics.

### Views on COVID-19 vaccine mandates

Nearly half (46%) of HCP believed that COVID-19 vaccination should be mandatory for HCP (Table 1). There was significant variation by HCP type, with mandatory vaccination most frequently supported by pediatricians (61%), followed by family medicine doctors (50%), then PAs/NPs/nurses (37%) and pharmacists (38%) (*p* < 0.01). Support for mandatory vaccination was also much more frequent among boosted HCP (55%) versus not boosted HCP (6%) (*p* < 0.01) (Supplementary Table 2), and among HCP in urban (52%) and suburban (45%) versus rural (39%) practices (*p* < 0.01) (Supplementary Table 5).

### Reasons for not vaccinating against COVID-19

**Fig. 1:**
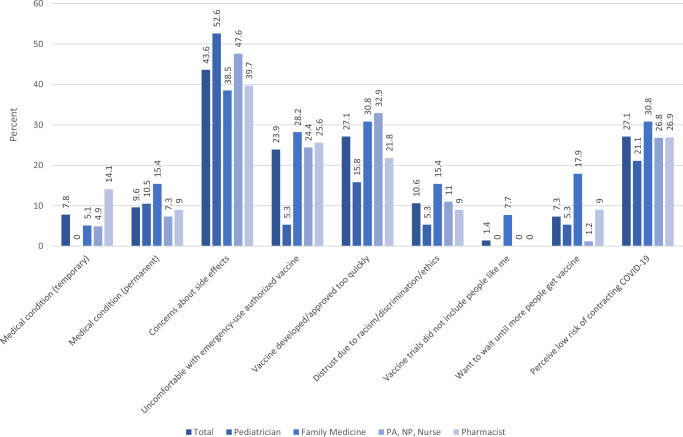
Reasons for not vaccinating among healthcare personnel not boosted against COVID-19 (*n* = 218). None of these reasons differed significantly (*p* < 0.05) by type of Healthcare Personnel, except for “vaccine trials did not include people like me” (*p* < 0.01) and “want to wait until more people get vaccine” (*p* = 0.01).

Among the 218 HCP who had not yet received a booster dose, the most common reason given for not vaccinating was concerns about side effects (44%) (Fig. 1). Other reasons included the speed of vaccine development and approval (27%), low perceived risk of infection (27%), discomfort with Emergency Use Authorization (EUA) (24%), distrust due to racism and previous unethical treatment of minorities (11%), permanent medical conditions (10%), temporary medical conditions (8%), wanting to wait until more are vaccinated (7%), and vaccine trials not including people like oneself (1%). Most of these reasons did not differ significantly by HCP type, except for “vaccine trials did not include people like me” (*p* < 0.01) and “want to wait until more people get vaccine” (*p* = 0.01), both of which were infrequently given, though most frequent among family medicine doctors.

### Vaccinating patients against COVID-19

Nearly three-quarters (73%) of HCP’s practices offered COVID-19 vaccines to their patients. The Pfizer-BioNTech vaccine was offered most frequently (86%), followed by Moderna (72%), J&J (12%), and Novavax (7%) (*p* < 0.01) (Table 1). Strategies commonly used to improve completion of the COVID-19 vaccine series included scheduling the next dose at the current visit (69%), paper reminder cards (46%), reminder telephone calls (39%), using a computerized immunization database/registry (39%), reminder text messages (34%), reminder emails (32%), and flagging patient charts (31%).

### Obstacles to vaccinating patients against COVID-19

**Fig. 2:**
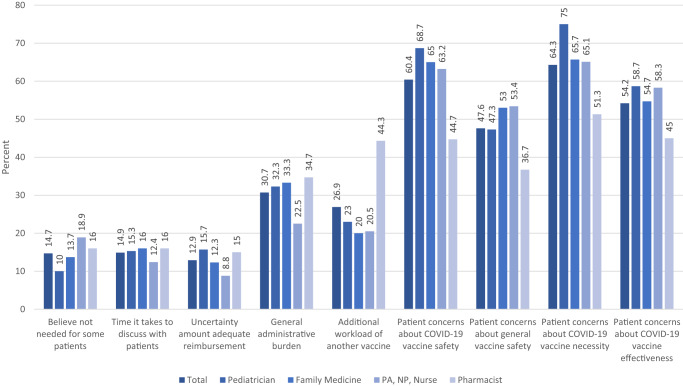
Obstacles to administering COVID-19 vaccines for healthcare personnel (*n* = 1207). All of these reasons differed significantly (*p* < 0.05) by type of Healthcare Personnel, except for “time it takes to discuss with patients” (*p* = 0.54).

More than half of HCP reported patient concerns as obstacles to vaccinating patients against COVID-19, whether about the necessity of the vaccine (64%), its safety (60%), or its effectiveness (54%) (Fig. 2). These patient concerns were reported most frequently among pediatricians and least frequently among pharmacists (*p* < 0.01). Boosted HCP were less likely than not boosted HCP to believe COVID-19 vaccines unnecessary for some patients (13% vs 23%, *p* < 0.01), yet more likely to report concerns among their patients about the necessity of COVID-19 vaccines (66% vs 58%, *p* = 0.03) (Supplementary Table 4).

### Strength of recommendations to patients to receive COVID-19 vaccines

**Fig. 3:**
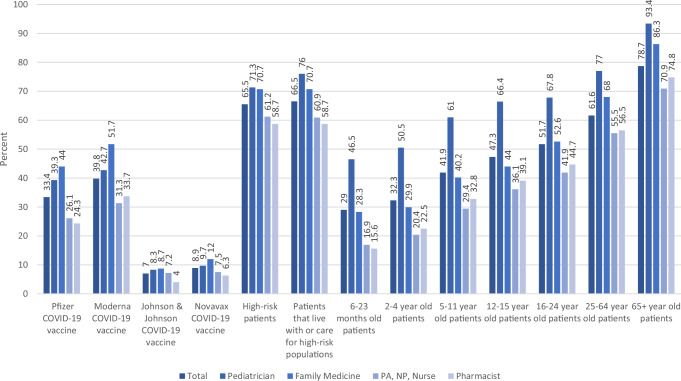
Strong recommendations by healthcare personnel (*n* = 1207) for specific populations to receive COVID-19 vaccine. All of these recommendations differed significantly (*p* < 0.05) by type of Healthcare Personnel, except for Johnson & Johnson COVID-19 vaccine (*p* = 0.10) and Novavax COVID-19 vaccine (*p* = 0.07).

One-third (33%) of HCP strongly recommended the Pfizer-BioNTech COVID-19 vaccine to their patients, compared to 40% for Moderna, 9% for Novavax, and 7% for J&J COVID-19 vaccines, respectively (*p* < 0.01) (Fig. 3). Family medicine doctors strongly recommended the Pfizer-BioNTech (44%) and Moderna (52%) vaccines most frequently, followed by pediatricians (39 and 43%, respectively), then PAs/NPs/nurses (26 and 31%, respectively) and pharmacists (24 and 34%, respectively) (*p* < 0.01). No significant difference by HCP type was found for strong recommendations for the J&J (*p* = 0.10) or Novavax vaccine (*p* = 0.07). Boosted HCP strongly recommended three of the four COVID-19 vaccines to their patients significantly more frequently than not boosted HCP: Pfizer-BioNTech (38% vs 12%; *p* < 0.01), Moderna (46% vs 11%; *p* < 0.01), and J&J (8% vs 2%; *p* < 0.01); no significant difference by booster status was found for strong recommendations of the Novavax vaccine (9% vs 6%; *p* = 0.16) (Supplementary Table 4).

Two-thirds of HCP strongly recommended COVID-19 vaccination to patients at high risk of severe COVID-19 (66%) and patients who were close contacts of high-risk persons (67%) (Fig. 3). HCP were less likely to strongly recommend COVID-19 vaccination to their younger patients than their older patients: 79% strongly recommended COVID-19 vaccination to patients at least 65 years old, compared to 62% for patients 25–64 years old, 52% for patients 16–24 years old, 47% for patients 12–15 years old, 42% for patients 5–11 years old, 32% for patients 2–4 years old, and 29% for patients 6–23 months old. Pediatricians strongly recommended COVID-19 vaccination most frequently to all aforementioned age groups, followed by family medicine doctors, then pharmacists and PAs/NPs/nurses (*p* < 0.01). Boosted HCP were more likely than not boosted HCP to strongly recommend COVID-19 vaccines to patients of all age groups (*p* < 0.01) (Supplementary Table 4). HCP in urban practices were more likely than HCP in rural practices to strongly recommend COVID-19 vaccines to patients of varying ages (Supplementary Table 7).

### Strength of recommendations to patients to receive routine vaccines

**Fig. 4:**
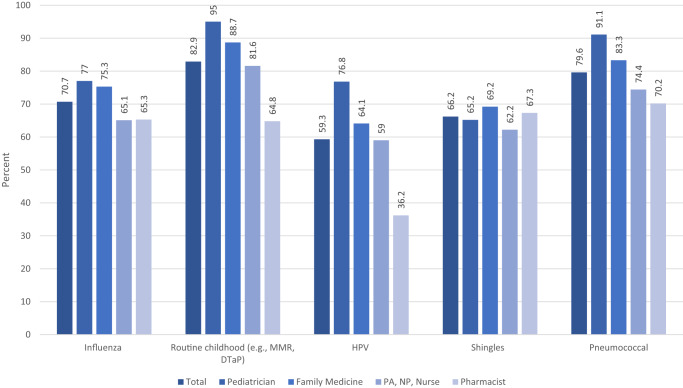
Strong recommendations by healthcare personnel (*n* = 1207) for eligible patients to receive routine vaccines. All of these recommendations differed significantly (*p* < 0.05) by type of Healthcare Personnel, except for shingles vaccine (*p* = 0.31).

HCP were most likely to strongly recommend routine childhood vaccines (e.g., MMR/DTaP) (83%) and pneumococcal vaccines (80%), followed by influenza vaccines (71%), shingles vaccines (66%), and HPV vaccines (59%) (Fig. 4). Pediatricians recommended these vaccines most frequently (except shingles vaccine, which is for older populations and thus not routinely administered by pediatricians). Pharmacists recommended routine vaccines least frequently (with the exception of shingles and influenza vaccines, which are both commonly given at pharmacies). Boosted HCP were more likely than not boosted HCP to strongly recommend routine vaccines to patients (*p* < 0.01; Supplementary Table 4).

### Impact of the pandemic on routine vaccination

Nearly all HCP (96%) had faced at least one obstacle to routine vaccination since the start of the pandemic (Table 3). The most common obstacle was decreased access to patients (e.g., fewer in-person visits), faced by two-thirds (66%) of HCP; interestingly, decreased access to patients was faced significantly more among boosted HCP (68%) than not boosted HCP (56%; *p* < 0.01; Supplementary Table 3). Other obstacles faced included disruption of vaccine supply (38%), staffing and personal protective equipment (PPE) shortages (37%), and lenient enforcement of school immunization requirements (28%). Staffing/PPE shortages and disruption of vaccine supply were experienced less frequently by pediatricians (22–27%) than by other HCP (36–48%) (*p* < 0.01). More than two-thirds of HCP anticipated facing each of these obstacles in the future.

**Table 3:** Impact of the pandemic on routine vaccination by type of healthcare personnel

	Total	Pediatrician	Family Medicine	PA, NP, Nurse	Pharmacist	*p*-value^a^
	*N* = 1207 (%)	*N* = 300 (%)	*N* = 300 (%)	*N* = 307 (%)	*N* = 300 (%)	
Included telehealth visits before March 2020	228 (18.9)	46 (15.3)	65 (21.7)	79 (25.7)	38 (12.7)	**<0.01**
% of total visits telehealth before March 2020						0.04
0–24%	204 (89.5)	44 (95.7)	63 (96.9)	67 (84.8)	30 (78.9)	–
25–49%	9 (3.9)	0 (0.0)	0 (0.0)	7 (8.9)	2 (5.3)	–
50–74%	10 (4.4)	2 (4.3)	1 (1.5)	3 (3.8)	4 (10.5)	–
75–100%	5 (2.2)	0 (0.0)	1 (1.5)	2 (2.5)	2 (5.3)	–
Included telehealth visits since March 2020	971 (80.4)	273 (91.0)	283 (94.3)	291 (94.8)	124 (41.3)	**<0.01**
% of total visits telehealth before March 2020						**<0.01**
0–24%	627 (64.6)	228 (83.5)	198 (70.0)	140 (48.1)	61 (49.2)	–
25–49%	256 (26.4)	37 (13.6)	67 (23.7)	108 (37.1)	44 (35.5)	–
50–74%	75 (7.7)	6 (2.2)	15 (5.3)	40 (13.7)	14 (11.3)	–
75–100%	13 (1.3)	2 (0.7)	3 (1.1)	3 (1.0)	5 (4.0)	–
Plan to continue telehealth after pandemic	846 (87.1)	220 (80.6)	256 (90.5)	261 (89.7)	109 (87.9)	**<0.01**
Decreased ability to vaccinate due to telehealth	487 (50.2)	126 (46.2)	139 (49.1)	164 (56.4)	58 (46.8)	0.08
Obstacles to vaccinating during pandemic so far:						
Decreased access to patients	790 (65.5)	233 (77.7)	205 (68.3)	195 (63.5)	157 (52.3)	**<0.01**
Lenient enforcement of school requirements	342 (28.3)	105 (35.0)	76 (25.3)	82 (26.7)	79 (26.3)	0.03
Disruption of vaccine supply	459 (38.0)	82 (27.3)	120 (40.0)	136 (44.3)	121 (40.3)	**<0.01**
Staffing and PPE shortages	442 (36.6)	66 (22.0)	108 (36.0)	125 (40.7)	143 (47.7)	**<0.01**
Obstacles to vaccinating expected in the future:						
Decreased access to patients	540 (68.4)	159 (68.2)	147 (71.7)	134 (68.7)	100 (63.7)	0.45
Lenient enforcement of school requirements	231 (67.5)	74 (70.5)	48 (63.2)	56 (68.3)	53 (67.1)	0.78
Disruption of vaccine supply	324 (70.6)	60 (73.2)	84 (70.0)	97 (71.3)	83 (68.6)	0.91
Staffing and PPE shortages	302 (68.3)	43 (65.2)	58 (53.7)	86 (68.8)	115 (80.4)	**<0.01**
More patients concerned about routine vaccines since pandemic	862 (71.4)	215 (71.7)	212 (70.7)	222 (72.3)	213 (71.0)	0.97
More patients refusing routine vaccines since pandemic	685 (56.8)	179 (59.7)	191 (63.7)	184 (59.9)	131 (43.7)	**<0.01**
Since March 2020, practice implemented changes to boost vaccination	648 (53.7)	145 (48.3)	137 (45.7)	148 (48.2)	218 (72.7)	**<0.01**
Patient-focused	530 (81.8)	112 (77.2)	105 (76.6)	121 (81.8)	192 (88.1)	0.02
Provider-focused	378 (58.3)	75 (51.7)	80 (58.4)	98 (66.2)	125 (57.3)	0.09
Practice-focused	381 (58.8)	87 (60.0)	77 (56.2)	84 (56.8)	133 (61.0)	0.76
Improved vaccine availability and access	347 (53.5)	57 (39.3)	58 (42.3)	86 (58.1)	146 (67.0)	**<0.01**
Practice stopped routine vaccines since March 2020	69 (10.9)	12 (8.3)	16 (11.9)	16 (11.3)	25 (11.8)	0.72

*PA* Physician Assistant, *NP* Nurse Practitioner.

^a^Boldface indicates statistical significance (*p* < 0.05) using Pearson’s *χ*^2^ Test.

More than half (54%) of HCP’s practices made changes during the pandemic in attempts to improve routine vaccination. Most of these practices incorporated patient-level interventions (82%); more than half incorporated interventions at the practice-level (59%) or provider-level (58%). More than half (54%) improved vaccine availability and access. Roughly 11% of practices were forced to stop or pause routine vaccination since the pandemic.

### Vaccine discussions with patients

HCP spent an average of 5.8 hours per week talking with patients about vaccines. Pharmacists spent the most time per week talking with patients about vaccines (7.4 hours), followed by pediatricians (5.9 hours), PAs/NPs/nurses (5.9 hours), and family medicine doctors (4.1 hours) (*p* < 0.01). More than half of HCP (59%) spent between 2 and 5 hours per week (Table 4). Nearly half (49%) of HCP reported that less than one quarter of their patients had vaccine concerns, though this varied greatly by type of HCP: 66% of pediatricians had few concerned patients, compared to 47% of pharmacists, 44% of PAs/NPs/nurses, and 40% of family medicine doctors (*p* < 0.01). Boosted HCP had fewer patients with vaccine concerns than HCP who were not boosted; 53% of boosted HCP had few concerned patients, compared to 33% of HCP who were not boosted (*p* < 0.01) (Supplementary Table 4). One-on-one conversation was by far the method most frequently reported by HCP as often being used to share vaccine information with their patients (86%), followed by posters/flyers/brochures in their office (34%), and their practice’s (23%) or other credible websites (24%); all remaining methods inquired about (e.g., email newsletter, their practice’s social media account) were often used by less than one-tenth of HCP (Table 4).

**Table 4:** Vaccine resources and discussions with patients by type of healthcare personnel

	Total	Pediatrician	Family Medicine	PA, NP, Nurse	Pharmacist	*p*-value^a^
	*N* = 1207 (%)	*N* = 300 (%)	*N* = 300 (%)	*N* = 307 (%)	*N* = 300 (%)	
Average hours per week spent talking with patients about vaccines						**<0.01**
0–1	182 (15.1)	30 (10.0)	69 (23.0)	52 (16.9)	31 (10.3)	–
2–3	439 (36.4)	112 (37.3)	125 (41.7)	102 (33.2)	100 (33.3)	–
4–5	275 (22.8)	78 (26.0)	60 (20.0)	72 (23.5)	65 (21.7)	–
6–9	79 (6.5)	21 (7.0)	14 (4.7)	23 (7.5)	21 (7.0)	–
10–19	151 (12.5)	41 (13.7)	25 (8.3)	32 (10.4)	53 (17.7)	–
20+	81 (6.7)	18 (6.0)	7 (2.3)	26 (8.5)	30 (10.0)	
Proportion of patients with vaccine concerns						**<0.01**
<25%	587 (49.2)	196 (66.0)	116 (39.5)	135 (44.0)	140 (47.3)	–
25–50%	416 (34.8)	77 (25.9)	114 (38.8)	111 (36.2)	114 (38.5)	–
51–75%	143 (12.0)	18 (6.1)	45 (15.3)	43 (14.0)	37 (12.5)	–
76–100%	48 (4.0)	6 (2.0)	19 (6.5)	18 (5.9)	5 (1.7)	–
Proportion of vaccination visits billed for administration						**<0.01**
<25%	180 (17.5)	25 (9.4)	40 (15.7)	70 (28.0)	45 (17.3)	–
25–50%	123 (11.9)	19 (7.2)	33 (12.9)	37 (14.8)	34 (13.1)	–
51–75%	80 (7.8)	19 (7.2)	15 (5.9)	22 (8.8)	24 (9.2)	–
76–100%	647 (62.8)	202 (76.2)	167 (65.5)	121 (48.4)	157 (60.4)	–
Proportion of vaccination visits billed for counseling						**<0.01**
<25%	691 (68.6)	115 (47.5)	204 (77.6)	166 (66.9)	206 (80.8)	–
25–50%	93 (9.2)	17 (7.0)	23 (8.7)	38 (15.3)	15 (5.9)	–
51–75%	69 (6.8)	18 (7.4)	16 (6.1)	15 (6.0)	20 (7.8)	–
76–100%	155 (15.4)	92 (38.0)	20 (7.6)	29 (11.7)	14 (5.5)	–
Often used for vaccine information:						
News media	140 (11.6)	25 (8.3)	53 (17.7)	32 (10.4)	30 (10.0)	**<0.01**
Social media	74 (6.1)	14 (4.7)	21 (7.0)	23 (7.5)	16 (5.3)	0.42
Private social media groups	65 (5.4)	12 (4.0)	28 (9.3)	16 (5.2)	9 (3.0)	**<0.01**
Email newsletters/listservs	242 (20.0)	51 (17.0)	47 (15.7)	52 (16.9)	92 (30.7)	**<0.01**
Websites	579 (48.0)	116 (38.7)	123 (41.0)	164 (53.4)	176 (58.7)	**<0.01**
Blogs	50 (4.1)	5 (1.7)	15 (5.0)	16 (5.2)	14 (4.7)	0.10
Message boards	68 (5.6)	7 (2.3)	19 (6.3)	25 (8.1)	17 (5.7)	**0.02**
Text message alerts	81 (6.7)	12 (4.0)	17 (5.7)	25 (8.1)	27 (9.0)	0.06
Podcasts	118 (9.8)	20 (6.7)	33 (11.0)	36 (11.7)	29 (9.7)	0.16
Publications in academic/medical journals	898 (74.4)	237 (79.0)	218 (72.7)	233 (75.9)	210 (70.0)	0.07
Trusted for vaccine information:						
News media	102 (8.5)	27 (9.0)	34 (11.3)	24 (7.8)	17 (5.7)	0.09
Social media	41 (3.4)	11 (3.7)	11 (3.7)	11 (3.6)	8 (2.7)	0.88
Academic/medical journals	1,052 (87.2)	277 (92.3)	264 (88.0)	264 (86.0)	247 (82.3)	**<0.01**
Academic/medical institutions	1,029 (85.3)	272 (90.7)	253 (84.3)	259 (84.4)	245 (81.7)	**0.02**
Professional medical organizations	1,023 (84.8)	273 (91.0)	248 (82.7)	256 (83.4)	246 (82.0)	**<0.01**
Other healthcare providers	707 (58.6)	185 (61.7)	178 (59.3)	178 (58.0)	166 (55.3)	0.46
Centers for Disease Control and Prevention (CDC)	933 (77.3)	265 (88.3)	219 (73.0)	231 (75.2)	218 (72.7)	**<0.01**
Food and Drug Administration (FDA)	886 (73.4)	247 (82.3)	215 (71.7)	213 (69.4)	211 (70.3)	**<0.01**
State and local public health departments	870 (72.1)	243 (81.0)	220 (73.3)	209 (68.1)	198 (66.0)	**<0.01**
Vaccine-focused non-profit organizations	532 (44.1)	142 (47.3)	136 (45.3)	131 (42.7)	123 (41.0)	0.41
Often used to share vaccine information with patients:						
One-on-one conversation	1,032 (85.5)	270 (90.0)	264 (88.0)	267 (87.0)	231 (77.0)	**<0.01**
Email newsletter	79 (6.5)	12 (4.0)	18 (6.0)	27 (8.8)	22 (7.3)	0.10
My social media accounts	51 (4.2)	5 (1.7)	18 (6.0)	13 (4.2)	15 (5.0)	0.054
Other credible social media accounts	57 (4.7)	5 (1.7)	15 (5.0)	18 (5.9)	19 (6.3)	**0.03**
My (or my practice’s) website	276 (22.9)	90 (30.0)	48 (16.0)	85 (27.7)	53 (17.7)	**<0.01**
Other credible websites	291 (24.1)	92 (30.7)	47 (15.7)	92 (30.0)	60 (20.0)	**<0.01**
Videos	98 (8.1)	10 (3.3)	29 (9.7)	33 (10.7)	26 (8.7)	**<0.01**
Posters/flyers/brochures in office	413 (34.2)	121 (40.3)	98 (32.7)	115 (37.5)	79 (26.3)	**<0.01**
It’s easy to stay up-to-date on vaccine recommendations, contraindications, controversies	812 (67.3)	231 (77.0)	203 (67.7)	199 (64.8)	179 (59.7)	**<0.01**
Patients sometimes ask vaccine questions to which you are unsure of the scientific answer	810 (67.1)	172 (57.3)	203 (67.7)	223 (72.6)	212 (70.7)	**<0.01**
It’d be helpful to know a patient’s vaccine intent and concerns prior to a visit	1,040 (86.2)	256 (85.3)	247 (82.3)	275 (89.6)	262 (87.3)	0.066
Feel well prepared for vaccine conversations with patients	1,047 (86.7)	280 (93.3)	266 (88.7)	260 (84.7)	241 (80.3)	**<0.01**
Have everything needed to share vaccine info with patients	922 (76.4)	246 (82.0)	222 (74.0)	241 (78.5)	213 (71.0)	**<0.01**
More information would help me recommend COVID-19 vaccines to my patients	192 (21.8)	39 (18.0)	44 (25.7)	48 (21.6)	61 (22.5)	0.32
Interest in a CME module on how to discuss COVID-19 and other vaccines with patients	472 (39.1)	108 (36.0)	109 (36.3)	132 (43.0)	123 (41.0)	0.20
Interest in online resource for HCP detailing how to talk with patients, vaccine recommendations, and vaccine safety issues	792 (65.6)	201 (67.0)	201 (67.0)	191 (62.2)	199 (66.3)	0.54
Interest in website to refer patients to that provides them regularly updated and individually tailored vaccine info	798 (66.1)	205 (68.3)	193 (64.3)	197 (64.2)	203 (67.7)	0.59
Adverse Event Reporting						
Familiar with VAERS	566 (93.7)	154 (99.4)	125 (86.2)	142 (91.6)	145 (97.3)	**<0.01**
Familiar with (fictitious) IARM system	475 (78.8)	121 (83.4)	118 (76.1)	120 (78.9)	116 (76.8)	0.41
Ever reported to VAERS	195 (34.5)	58 (37.7)	34 (27.2)	42 (29.6)	61 (42.1)	**0.03**
Ever reported to (fictitious) IARM system	120 (25.3)	39 (32.2)	21 (17.8)	24 (20.0)	36 (31.0)	**0.02**

*PA* Physician Assistant, *NP* Nurse Practitioner, *VAERS* Vaccine Adverse Event Reporting System, *IARM* Immunization Adverse Reaction Monitoring (fictitious).

^a^Boldface indicates statistical significance (*p* < 0.05) using Pearson’s *χ*^2^ Test.

### Sources of vaccine information

Nearly three-quarters (74%) of HCP often used publications in academic/medical journals for vaccine information (Table 4). Nearly half (48%) often used websites, and one-fifth (20%) often used email newsletters. All remaining sources inquired about were often used by less than one-tenth of HCP. Academic/medical journals (87%) and institutions (85%), along with professional medical organizations (85%), were the most frequently trusted sources of vaccine information among HCP. About three-quarters of HCP trusted the CDC (77%), the FDA (73%), and state/local public health departments (72%) for vaccine information. Only 9% of HCP trusted the news media, and only 3% trusted social media. All these sources of vaccine information were more frequently trusted by boosted HCP versus HCP who were not boosted (*p* < 0.01) (Supplementary Table 4).

Two-thirds (67%) of HCP found it easy to stay up-to-date on vaccine recommendations, contraindications, and controversies; boosted HCP found this easy more frequently (69%) than HCP who were not boosted (58%) (*p* < 0.01). Pediatricians found it easiest to stay up-to-date (77%), followed by family medicine doctors (68%), PAs/NPs/nurses (65%), and pharmacists (60%) (Table 4). Two-thirds (67%) of HCP also reported that their patients sometimes asked them vaccine questions to which they were unsure of the scientific answer; PAs/NPs/nurses reported this most frequently (73%), followed by pharmacists (71%), family medicine doctors (68%), and pediatricians (57%). More than three-quarters (76%) of HCP perceived having everything they needed to share vaccine information with their patients; pediatricians perceived having what they needed most frequently (82%), followed by PAs/NPs/nurses (79%), family medicine doctors (74%), and pharmacists (71%). However, nearly one-quarter (22%) of HCP felt more information would help them recommend COVID-19 vaccines to their patients, and many were interested in resources to improve their vaccine discussions with patients such as a continuing medical education (CME) module (39%) or an online resource/website (66%).

### Vaccine billing

Nearly two-thirds (63%) of HCP billed for administration for at least three quarters of their vaccination visits, though this varied greatly by type of HCP: 76% of pediatricians billed for administration for most vaccination visits, compared to 66% of family medicine doctors, 60% of pharmacists, and 48% of PAs/NPs/nurses (*p* < 0.01) (Table 4). Conversely, more than two-thirds (69%) of HCP only billed for counseling for less than one quarter of their vaccination visits, though this also varied greatly by type of HCP: 48% of pediatricians billed for counseling for few vaccination visits, compared to 67% of PAs/NPs/nurses, 78% of family medicine doctors, and 81% of pharmacists (*p* < 0.01).

### Familiarity with vaccine adverse event reporting

Nearly all (94%) HCP asked about the CDC’s Vaccine Adverse Event Reporting System (VAERS) said they were familiar with the system, of which more than one-third (35%) claimed they had made a report to VAERS at least once (Table 4). However, more than three-quarters (79%) of HCP asked about the fictitious IARM system said they were familiar with IARM, of which one-quarter (25%) claimed to have made a report to IARM at least once. Pediatricians (99%) and pharmacists (97%) were familiar with VAERS the most frequently, followed by PAs/NPs/nurses (92%) and family medicine doctors (86%) (*p* < 0.01). Of those familiar with VAERS, pharmacists (42%) and pediatricians (38%) also made reports to VAERS the most frequently, followed by PAs/NPs/nurses (30%) and family medicine doctors (27%) (*p* < 0.01). Those claiming familiarity with IARM did not differ significantly by HCP type (*p* = 0.41).

### Changes over time

Responses to several notable survey items showed highly significant changes among HCP between the September 2021 and January 2023 survey waves. Coverage with at least one dose of COVID-19 vaccine increased from 92 to 96% (*p* < 0.01) (Table 5); this was driven by a 3% increase among pediatricians (*p* = 0.01) and a 6% increase among PAs/NPs/nurses (*p* = 0.01) (Supplementary Table 8). The proportion of HCP practices providing specific COVID-19 vaccines increased from 77 to 86% for Pfizer (*p* < 0.01) and from 67 to 73% for Moderna (*p* < 0.01), and decreased from 27 to 12% for J&J (*p* < 0.01). Recommendations to vaccinate against COVID-19 decreased substantially (*p* < 0.01), regardless of vaccine type or specific patient population, and most notably among pharmacists. Recommendations to receive other vaccines also decreased, including for routine childhood vaccines (e.g., MMR, DTaP) (absolute 2% decrease), influenza (4% decrease), HPV (5% decrease), and shingles (4% decrease) (all *p* < 0.05). The proportion of HCP who had regularly taken care of patients with COVID-19 increased from 70 to 89% (*p* < 0.01), with significant increases among all four HCP types (*p* < 0.01). Support for COVID-19 vaccine mandates for HCP decreased from 65 to 46% (*p* < 0.01), with significant decreases among all four HCP types (*p* < 0.01). The proportion of HCP with high trust in CDC decreased from 79 to 73% (*p* < 0.01), most notably among pharmacists, among whom trust decreased by 10% (*p* < 0.01).

**Table 5:** Changes in survey responses between September 2021 and January 2023

Survey items	% Sept 2021	% Jan 2023	% Change	*P*-value^a^
High Trust in CDC^b^	79	73	−6	**<0.01**
Included telehealth visits before March 2020	15	19	3	**0.03**
Included telehealth visits since March 2020	78	80	2	0.25
Plan to continue telehealth after pandemic	83	87	4	**0.01**
Decreased ability to provide routine vaccination due to telehealth	51	50	−1	0.61
Practice currently administers vaccines	96	97	1	0.19
Practice participates in the Vaccines for Children (VFC) program	51	52	2	0.48
Since March 2020, practice implemented changes to boost vaccination	55	54	−1	0.49
Patient-focused	82	82	0	0.93
Provider-focused	50	58	8	**<0.01**
Practice-focused	54	59	5	0.09
Improved vaccine availability and access	45	54	9	**<0.01**
Practice stopped routine vaccines since March 2020	14	11	−3	0.17
Practice provided seasonal influenza vaccination: 2019–2020	94	92	−2	0.14
Practice provided seasonal influenza vaccination: 2020–2021	93	94	1	0.32
Obstacles to vaccinating during pandemic so far:				
Decreased access to patients	72	65	−7	**<0.01**
Lenient enforcement of school immunization requirements	24	28	5	**0.01**
Disruption of vaccine supply	23	38	15	**<0.01**
Disruption of vaccination due to the staffing and PPE shortages	31	37	6	**<0.01**
Obstacles to vaccinating expected in the future:				
Decreased access to patients	85	68	−17	**<0.01**
Lenient enforcement of school immunization requirements	63	68	4	0.26
Disruption of vaccine supply	67	71	4	0.33
Staffing and PPE shortages	63	68	6	0.10
Adverse Event Reporting				
Familiar with VAERS	93	94	1	0.55
Familiar with (fictitious) IARM system	73	79	6	**0.02**
Ever reported to VAERS	37	34	−2	0.45
Ever reported to (fictitious) IARM system	25	25	0	0.93
Received at least one COVID-19 vaccine	92	96	3	**<0.01**
Reasons for not vaccinating among HCP not vaccinated/boosted against COVID-19:				
Medical condition, temporary	17	8	−9	**0.02**
Medical condition, permanent	10	10	0	0.95
Concern, side effects	47	44	−3	0.61
Uncomfortable emergency-use authorized vaccine	30	24	−6	0.31
Vaccine developed/approved too quickly	33	27	−6	0.29
Distrust due to racism/discrimination/ethics	15	11	−4	0.31
Vaccine trials did not include people like me	4	1	−2	0.20
Want to wait until more people get vaccine	26	7	−19	**<0.01**
I have low risk for contracting COVID-19	30	27	−3	0.66
Obstacles to administering COVID-19 vaccines:				
Believe not needed for some patients	27	15	−12	**<0.01**
Time it takes to discuss with patients	21	15	−6	0.14
Uncertainty amount adequate reimbursement	21	13	−8	**0.04**
General administrative burden	27	31	3	0.51
Additional workload of another vaccine	30	27	−3	0.60
Patient concerns about COVID-19 vaccine safety	59	60	1	0.84
Patient concerns about general vaccine safety	59	48	−12	**0.04**
Patient concerns about COVID-19 vaccine necessity	57	64	8	0.17
Patient concerns about COVID-19 vaccine effectiveness	56	54	−1	0.81
COVID-19 vaccination should be mandated for healthcare workers	65	46	−18	**<0.01**
Regularly taken care of COVID-19 patients	70	89	18	**<0.01**
COVID-19 vaccine recommendations for specific patient populations				
High-risk patients	87	82	−5	**<0.01**
Patients that live with or care for high-risk persons	90	83	−7	**<0.01**
12–15-year-old patients	73	66	−7	**<0.01**
16–24-year-old patients	82	70	−12	**<0.01**
25–64-year-old patients	89	80	−9	**<0.01**
65+ year-old patients	94	90	−4	**<0.01**
12–15-year-old high-risk patients	85	80	−5	**<0.01**
16–24-year-old high-risk patients	89	83	−6	**<0.01**
25–64-year-old high-risk patients	93	88	−5	**<0.01**
65+ year-old high-risk patients	95	92	−4	**<0.01 **
COVID-19 vaccine recommendations by vaccine				
Pfizer	86	55	−31	**<0.01**
Moderna	84	60	−23	**<0.01**
Johnson & Johnson	44	15	−30	**<0.01**
Routinely co-administering COVID-19 vaccines with other recommended vaccines	61	62	0	0.86
Practice provides COVID-19 vaccines	67	73	6	**<0.01**
Pfizer	77	86	9	**<0.01**
Moderna	65	72	7	**<0.01**
Johnson & Johnson	27	12	−15	**<0.01**
Strategies used to improve COVID-19 vaccine series completion:				
Paper-based reminder card	60	46	−13	**<0.01**
Reminder telephone calls	54	39	−14	**<0.01**
Flagging patient charts	33	31	−2	0.47
Scheduling next dose at current visit	83	69	−14	**<0.01**
Computerized immunization database/registry	51	39	−12	**<0.01**
More information would help me recommend COVID-19 vaccines to my patients	26	22	−4	0.08
Routine vaccine recommendations for eligible patients by vaccine				
Influenza	93	89	−4	**<0.01**
Routine childhood (e.g., MMR, DTaP)	94	91	−2	**0.04**
HPV	84	80	−5	**0.01**
Shingles	92	87	−4	**<0.01**
Pneumococcal	95	94	−1	0.37
Interest in a CME module on how to discuss COVID-19 and other vaccines with patients	46	39	−7	**<0.01**
Interest in online resource for HCP detailing how to talk with patients, vaccine recommendations, and vaccine safety issues	46	66	19	**<0.01**

*CDC* Centers for Disease Control and Prevention, *VAERS* Vaccine Adverse Event Reporting System, *IARM* Immunization Adverse Reaction Monitoring (fictitious).

^a^Boldface indicates statistical significance (*p* < 0.05) using Pearson’s *χ*^2^ Test.

^b^see Supplementary Table 1.

Plans to continue telehealth after the pandemic increased from 83 to 87% (*p* = 0.01). Provider-focused interventions to improve routine vaccination increased from 50 to 58% (*p* < 0.01), most notably among pediatricians, among whom implementation increased by 13% (*p* = 0.03). Interventions to improve vaccine availability/access also increased from 45 to 54% (*p* < 0.01). Increases in the proportion of HCP facing obstacles to vaccinating during the pandemic included: disruption of vaccine supply (23 to 38%, *p* < 0.01), staffing and PPE shortages (31 to 37%, *p* < 0.01), and lenient enforcement of school immunization requirements (24 to 28%, *p* = 0.01). However, the proportion of HCP reporting decreased access to patients as an obstacle decreased from 72 to 65% (*p* < 0.01), and of those, the proportion expecting access to remain an obstacle in the future decreased from 85 to 68% (*p* < 0.01). The only reasons for not vaccinating that declined significantly over time were temporary medical conditions (17 to 8%, *p* = 0.02) and wanting to wait until more people get vaccinated (26 to 7%, *p* < 0.01). The only obstacles to administering COVID-19 vaccines that declined significantly over time were uncertainty amount adequate reimbursement (21 to 13%, *p* = 0.04), belief that COVID-19 vaccines are not needed for some patients (27 to 15%, *p* < 0.01), and patient concerns about general vaccine safety (59 to 48%, *p* = 0.04); the declines in uncertainty amount adequate reimbursement and patient concerns about general vaccine safety were driven largely by decreases among family medicine doctors of 29% (*p* < 0.01) and 30% (*p* = 0.04), respectively. Use of strategies to improve COVID-19 vaccine series completion (e.g., paper-based reminder card, reminder telephone calls, scheduling next dose at current visit, computerized immunization database/registry) decreased. Although interest in a CME module on how to discuss COVID-19 and other vaccines with patients decreased from 46 to 39% (*p* < 0.01), interest in an online resource on the same topic increased from 46 to 66% (*p* < 0.01), with significant increases among all four HCP types (*p* < 0.01).

## Discussion

In this January 2023 panel survey, more than 8 out of 10 HCP had received a COVID-19 booster dose. Boosted HCP recommended vaccination to their patients more than not boosted HCP. HCP were most likely to recommend COVID-19 vaccination to patients who were older or high-risk (or in close contact with high-risk persons). Pediatricians had the highest vaccine coverage and strongest vaccine recommendations, followed by family medicine doctors, pharmacists, and PAs/NPs/nurses. Nearly half of not boosted HCP were concerned about potential side effects, and about one-quarter were concerned the vaccine was developed and approved too quickly, perceived a low risk of infection, or were uncomfortable with EUA, reflecting prevalent attitudes in the general population at this time^4^.

The most trusted sources of vaccine information among HCP were academic/medical journals and institutions and professional medical organizations, though about three-quarters of HCP also trusted government institutions such as CDC, FDA, and state/local public health departments. Trust in CDC was strongly positively associated with being boosted against COVID-19, and was most common among pediatricians, followed by family medicine doctors, PAs/NPs/nurses, and pharmacists. A strong relationship between COVID-19 vaccination and trust in CDC has also been seen among the general population^4,6,12^; a nationally representative panel survey of US adults, conducted in September 2022, found those with high trust in CDC had seven times greater odds of being boosted than those with low trust^4^. However, trust in CDC has declined over the course of the pandemic^13^.

Though HCP spent almost six hours per week talking with patients about vaccines on average, much of this time was uncompensated. This illustrates the burden on HCP to dedicate substantial time and energy to something for which they are not fully compensated or trained. And these reported hours do not count the time and energy spent outside of these conversations themselves; one-third of HCP found it difficult to stay up-to-date on vaccine recommendations and controversies, and two-thirds confessed patients asked vaccine questions to which they were unsure of the answer, both presumably requiring additional independent preparation and research. Pharmacists, PAs/NPs/nurses, and family medicine doctors all reported these challenges more frequently than pediatricians. These findings are supported by the literature: HCP have cited a lack of time as the greatest barrier to their vaccine discussions with patients, which often leads to nurses spending more time on such discussions than pediatricians or family medicine doctors^14^; and despite pediatric and family medicine doctors recognizing the importance of vaccine communication in their work, training in vaccine communication is often not included in medical school curriculum or residency programs^15–17^.

Although COVID-19 vaccine coverage among HCP increased slightly between September 2021 and January 2023, the proportion of HCP recommending vaccines (COVID-19 and routine) decreased substantially among nearly all vaccine types and specific patient populations, as did trust in CDC and support for HCP COVID-19 vaccine mandates (despite some evidence for their effectiveness)^18^. This is concerning as HCP have long been the most frequently used and credible source of vaccine information^5^, especially given public suspicion of vaccine information from pharmaceutical companies and government^4^, and encouragement from HCP is strongly associated with patients vaccinating against COVID-19^6^. Unvaccinated HCP are unsurprisingly far less likely to recommend vaccinating to their patients^10^, but even vaccinated HCP need regularly updated resources to confidently discuss vaccines with hesitant patients, especially as vaccine science and public concerns continue to evolve. HCP interest in additional online resources to improve their discussions of COVID-19 and other vaccines with their patients also increased substantially between September 2021 and January 2023. Public health organizations should therefore prioritize providing online resources to HCP (especially pharmacists, PAs/NPs/nurses, and family medicine doctors) from trusted medical journals/institutions/organizations, to improve both their own vaccine decision-making and their support of their patients’ decision-making.

It is difficult to compare our data to other concurrent data, as the data on COVID-19 vaccine coverage from the winter of 2022–2023 that is currently publicly available does not stratify by HCP and largely focuses on the updated bivalent booster at the expense of the original monovalent booster. The CDC simplified their recommendations in April 2023 so that everyone at least 6 years of age is considered up-to-date if they have received at least one dose of the bivalent COVID-19 vaccine^19–21^. However, at the time of this survey (December 2022 to January 2023), up-to-date was still largely considered to mean fully vaccinated (with a primary series) and boosted, without specifying that the booster should be the most recent version, since the new bivalent booster had just been authorized in August 2022 and was recommended for adults only at least two months after their last dose and at least three months after a COVID-19 infection^22^. Some HCP we categorized as boosted may have received the bivalent booster, and some may have only received the monovalent booster; we did not require them to specify. The CDC no longer reports vaccine coverage for those receiving any booster dose, specifying only those who have received the updated bivalent booster dose. As of May 10, 2023, only about one-fifth (21%) of US adults had received the bivalent booster per CDC^23^. The Kaiser Family Foundation (KFF) estimated that 28% of US adults had received the bivalent booster as of January 2023^24^. Although we expect vaccine coverage to be substantially higher among HCP than the general population, that likely does not explain the entirety of the gap in reported booster coverage between our sample of HCP and other national sources. Conversely, a nationally panel survey conducted in September 2022 (immediately following authorization of the new bivalent boosters)^22^ found that nearly two-thirds (63%) of US adults had received a monovalent booster dose^4^, a gap that is much more realistically explained by traditionally higher vaccine coverage among HCP.

Another limitation of this study is that it was only powered to confirm differences between four strata of HCP. In focusing on the most common HCP types, specialists were naturally excluded in favor of primary care providers (PCPs). Thus, our findings may not be representative of specialists, who may have more influence on certain older populations than PCPs. We also combined PAs, NPs, and nurses into a single group, despite differing roles and education. However, a secondary analysis performed after our first survey among HCP that examined PAs/NPs separately from nurses found their vaccine coverage similar and supported combining them as the study was designed to do^10^. Our data also rely on self-reporting. More than three-quarters of our sample claimed familiarity with a fictitious adverse event monitoring system, which shows the potential effects of social desirability bias. Strengths of our work include our comparison of different types of HCP, the use of a well-established panel, and the ability to compare responses to many survey items at two timepoints^25^.

Although most HCP are vaccinated and boosted against COVID-19 and strongly recommend their patients vaccinate, the strength of HCP recommendations (for both COVID-19 and other routine vaccines) has dwindled over the course of the pandemic, along with trust in CDC. HCP averaged about six hours per week talking with patients about vaccines, much of which was uncompensated. Additional regularly updated online resources from trusted medical sources that clarify progressing science and address dynamic public concerns are needed to improve vaccine confidence among HCP and help them support their patients’ decision-making.

## Methods

### Recruitment

From December 20, 2022, to January 25, 2023, we recruited family medicine doctors, pediatricians, pharmacists, physician assistants, nurse practitioners, and nurses (including registered nurses and licensed practical nurses) who provided care directly to patients, using SurveyHealthcareGlobus^25^. SurveyHealthcareGlobus, a double opt-in network panel, comes from a population of more than 2 million people and is regularly validated and updated from standard core sources including medical and hospital directories, verified healthcare websites, and the American Medical Association. Recruitment only ceased after at least 300 HCP of each of the four main categories (pediatricians, family medicine doctors, PAs/NPs/nurses, and pharmacists) were surveyed. Respondents were given a $10 USD honorarium. Participants were members of the SurveyHealthcareGlobus panel and gave their consent to be surveyed. The Johns Hopkins Bloomberg School of Public Health Institutional Review Board considered this study public health surveillance and not human subject research.

### Survey content

This survey replicated much of the previous (September 2021) survey^10^, collecting data on: patient population (e.g., age, race/ethnicity, insurance), practice characteristics (e.g., type, size, location), COVID-19 vaccination status (both primary series and boosters), COVID-19 vaccine intention (among unvaccinated), reasons for not vaccinating against COVID-19 (among both unvaccinated and those vaccinated but not boosted), obstacles to vaccinating patients against COVID-19, strength of recommendations for COVID-19 as well as other vaccines, and the pandemic’s impact on routine vaccination practices. This survey also assessed familiarity with and use of Vaccine Adverse Event Reporting Systems (VAERS); to explore the effects of social desirability bias, respondents were randomized to receive questions about either the Food and Drug Administration and CDC’s Vaccine Adverse Event Reporting System^26^ or the fabricated Immunization Adverse Reaction Monitoring (IARM) system. Trust in CDC was again measured as a construct using a 14-item scale^27^. New items were added to this survey to explore vaccine resources and discussions with patients.

### Data analyses

Data were analyzed using Stata (version 16)^28^. Survey responses were summarized and stratified by the four main HCP type categories (pediatricians, family medicine doctors, PAs/NPs/nurses, and pharmacists), as well as whether the HCP had received at least one booster dose against COVID-19 and whether the practice location of the HCP was urban, suburban, or rural. As in the analysis of the previous survey^10^, we combined PAs, NPs, and nurses into one HCP type category to simplify comparisons and improve statistical power. Items included in both survey waves (September 2021 and January 2023) were compared to calculate the changes in response frequency over time. Differences were tested for statistical significance (*p* < 0.05) using Pearson’s *χ*^2^ Test, both within each HCP type and combined.

A composite, linear score was generated for the trust in CDC construct scale (Supplementary Table 1). The numerator was the sum of responses to all answered items within the scale (each 4-point Likert scale response was scored 0–3). The denominator was the total possible score (accounting for missing data), thus creating a scale from 0 to 100 (0 being consistent strong disagreement and 100 being consistent strong agreement). Cronbach’s alpha was estimated as 0.91, showing the scale’s strong reliability^29^. The score was dichotomized at the middle (50) to improve interpretability and facilitate comparisons over time, creating “high trust” and “low trust” groups.

We conducted multivariable logistic regression to assess whether receiving at least one booster dose against COVID-19 (a binary dependent variable) was associated with HCP type (a categorical independent variable) or trust in CDC (a dichotomous independent variable). Using the *logistic* command, odds ratios and 95% confidence intervals (CIs) were obtained for HCP being boosted versus not. We calculated both crude and adjusted ORs and CIs, to control for potential confounding of sociodemographic characteristics shown to be associated with COVID-19 booster status in this analysis (Supplementary Table 2) and previously^4^, such as graduation year (to approximate age), race/ethnicity, region, and practice location (urban/suburban/rural). Given the small numbers in each strata, we took a conservative approach and ran 100 bootstrap replications to calculate standard errors.

### Reporting summary

Further information on research design is available in the Nature Research Reporting Summary linked to this article.

## Supplementary information

Supplementary Tables

REPORTING SUMMARY

## Data Availability

Deidentified individual participant data will not be made available.
