# Author Correction: Changes in vaccine attitudes and recommendations among US Healthcare Personnel during the COVID-19 pandemic

**DOI:** 10.1038/s41541-025-01108-x

**Published:** 2025-03-28

**Authors:** Matthew Z. Dudley, Holly B. Schuh, Amanda Forr, Jana Shaw, Daniel A. Salmon

**Affiliations:** 1 Institute for Vaccine Safety, Johns Hopkins Bloomberg School of Public Health, Baltimore, MD USA; 2 Department of International Health, Johns Hopkins Bloomberg School of Public Health, Baltimore, MD USA; 3 Department of Epidemiology, Johns Hopkins Bloomberg School of Public Health, Baltimore, MD USA; 4 Element A LLC, Hershey, PA USA; 5 Division of Infectious Diseases, Department of Pediatrics, SUNY Upstate Medical University, Syracuse, NY USA; 6 Department of Health, Behavior and Society, Johns Hopkins Bloomberg School of Public Health, Baltimore, MD USA

Correction to: *npj Vaccines* 10.1038/s41541-024-00826-y, published online 28 February 2024

In the original version of this Article, the images for Figs. 1 and 3 were mistakenly switched (though the titles, footnotes, and text references for each Figure were correct). The authors apologize for this error and state that this does not change the scientific conclusions of the article. The original article has been corrected.

