# Supplementary material for: Changes in vaccine attitudes and recommendations among US Healthcare Personnel during the COVID-19 pandemic
Source: NPJ Vaccines. 2024 Feb 28;9:49. doi: 10.1038/s41541-024-00826-y (PMC10901873; doi:10.1038/s41541-024-00826-y)
Supplement: Supplementary file 1 — Supplementary Tables [file 41541_2024_826_MOESM1_ESM.pdf]

Supplementary Table 1. ***Composition and Properties of the Construct Scale Measuring Trust in the Centers for Disease Control and Prevention (CDC) by Type of Healthcare Personnel***

	Total N=1,207	Pediatrician N=300	Family Medicine N=300	PA, NP, Nurse[a] N=307	Pharmacist N=300	p-value[b]
High trust in CDC, n (%) [c]	883 (73.2%)	244 (81.3%)	214 (71.3%)	216 (70.4%)	209 (69.7%)	**<0.01**
Cronbach alpha (average interitem covariance) [d]	0.91 (0.23)	0.90 (0.21)	0.91 (0.25)	0.90 (0.22)	0.90 (0.21)	
The CDC does everything they should to protect the health of the population						0.16
Strongly disagree	62 (5.1%)	9 (3.0%)	17 (5.7%)	19 (6.2%)	17 (5.7%)	
Disagree	186 (15.4%)	39 (13.0%)	46 (15.3%)	60 (19.5%)	41 (13.7%)	
Agree	690 (57.2%)	172 (57.3%)	174 (58.0%)	166 (54.1%)	178 (59.3%)	
Strongly agree	269 (22.3%)	80 (26.7%)	63 (21.0%)	62 (20.2%)	64 (21.3%)	
The CDC are partly responsible for the illegal drug problems in this country [c]						**<0.01**
Strongly agree	71 (5.9%)	11 (3.7%)	23 (7.7%)	20 (6.5%)	17 (5.7%)	
Agree	270 (22.4%)	51 (17.0%)	64 (21.3%)	69 (22.5%)	86 (28.7%)	
Disagree	625 (51.8%)	150 (50.0%)	146 (48.7%)	176 (57.3%)	153 (51.0%)	
Strongly disagree	241 (20.0%)	88 (29.3%)	67 (22.3%)	42 (13.7%)	44 (14.7%)	
The CDC uses resources well						**0.02**
Strongly disagree	56 (4.6%)	6 (2.0%)	22 (7.3%)	16 (5.2%)	12 (4.0%)	
Disagree	266 (22.0%)	54 (18.0%)	62 (20.7%)	75 (24.4%)	75 (25.0%)	
Agree	760 (63.0%)	199 (66.3%)	187 (62.3%)	186 (60.6%)	188 (62.7%)	
Strongly agree	125 (10.4%)	41 (13.7%)	29 (9.7%)	30 (9.8%)	25 (8.3%)	
The CDC wastes money on health problems [c]						**<0.01**
Strongly agree	77 (6.4%)	13 (4.3%)	29 (9.7%)	14 (4.6%)	21 (7.0%)	
Agree	300 (24.9%)	59 (19.7%)	69 (23.0%)	77 (25.1%)	95 (31.7%)	
Disagree	654 (54.2%)	175 (58.3%)	162 (54.0%)	175 (57.0%)	142 (47.3%)	
Strongly disagree	176 (14.6%)	53 (17.7%)	40 (13.3%)	41 (13.4%)	42 (14.0%)	
The CDC keeps trying the same things to help the public, even when they don't work very well [c]						0.18
Strongly agree	111 (9.2%)	22 (7.3%)	40 (13.3%)	26 (8.5%)	23 (7.7%)	
Agree	520 (43.1%)	121 (40.3%)	129 (43.0%)	132 (43.0%)	138 (46.0%)	
Disagree	516 (42.8%)	140 (46.7%)	117 (39.0%)	137 (44.6%)	122 (40.7%)	
Strongly disagree	60 (5.0%)	17 (5.7%)	14 (4.7%)	12 (3.9%)	17 (5.7%)	
The CDC comes up with new ideas to solve health problems						0.26

Strongly disagree	44 (3.6%)	7 (2.3%)	12 (4.0%)	16 (5.2%)	9 (3.0%)	
Disagree	307 (25.4%)	69 (23.0%)	72 (24.0%)	75 (24.4%)	91 (30.3%)	
Agree	748 (62.0%)	191 (63.7%)	187 (62.3%)	191 (62.2%)	179 (59.7%)	
Strongly agree	108 (8.9%)	33 (11.0%)	29 (9.7%)	25 (8.1%)	21 (7.0%)	
The CDC bases recommendations on the best available science						**<0.01**
Strongly disagree	59 (4.9%)	9 (3.0%)	16 (5.3%)	20 (6.5%)	14 (4.7%)	
Disagree	193 (16.0%)	44 (14.7%)	40 (13.3%)	50 (16.3%)	59 (19.7%)	
Agree	623 (51.6%)	135 (45.0%)	161 (53.7%)	163 (53.1%)	164 (54.7%)	
Strongly agree	332 (27.5%)	112 (37.3%)	83 (27.7%)	74 (24.1%)	63 (21.0%)	
The CDC are not always able to help the health of the public [c]						**0.02**
Strongly agree	153 (12.7%)	33 (11.0%)	53 (17.7%)	33 (10.7%)	34 (11.3%)	
Agree	755 (62.6%)	188 (62.7%)	190 (63.3%)	196 (63.8%)	181 (60.3%)	
Disagree	247 (20.5%)	59 (19.7%)	46 (15.3%)	69 (22.5%)	73 (24.3%)	
Strongly disagree	52 (4.3%)	20 (6.7%)	11 (3.7%)	9 (2.9%)	12 (4.0%)	
The CDC are more concerned about some racial and ethnic groups than other groups [c]						**<0.01**
Strongly agree	97 (8.0%)	16 (5.3%)	34 (11.3%)	25 (8.1%)	22 (7.3%)	
Agree	303 (25.1%)	63 (21.0%)	71 (23.7%)	89 (29.0%)	80 (26.7%)	
Disagree	619 (51.3%)	153 (51.0%)	149 (49.7%)	160 (52.1%)	157 (52.3%)	
Strongly disagree	188 (15.6%)	68 (22.7%)	46 (15.3%)	33 (10.7%)	41 (13.7%)	
The CDC are concerned about all people, without caring about who has more or less money						**<0.01**
Strongly disagree	61 (5.1%)	5 (1.7%)	15 (5.0%)	26 (8.5%)	15 (5.0%)	
Disagree	243 (20.1%)	41 (13.7%)	57 (19.0%)	78 (25.4%)	67 (22.3%)	
Agree	657 (54.4%)	168 (56.0%)	166 (55.3%)	156 (50.8%)	167 (55.7%)	
Strongly agree	246 (20.4%)	86 (28.7%)	62 (20.7%)	47 (15.3%)	51 (17.0%)	
The CDC accurately informs the public of both health risks and benefits of medicines						**<0.01**
Strongly disagree	62 (5.1%)	8 (2.7%)	17 (5.7%)	20 (6.5%)	17 (5.7%)	
Disagree	229 (19.0%)	37 (12.3%)	51 (17.0%)	66 (21.5%)	75 (25.0%)	
Agree	703 (58.2%)	180 (60.0%)	182 (60.7%)	175 (57.0%)	166 (55.3%)	
Strongly agree	213 (17.6%)	75 (25.0%)	50 (16.7%)	46 (15.0%)	42 (14.0%)	
The CDC quickly helps the public with health problems						**<0.01**

Strongly disagree	64 (5.3%)	8 (2.7%)	21 (7.0%)	21 (6.8%)	14 (4.7%)	
Disagree	310 (25.7%)	71 (23.7%)	68 (22.7%)	78 (25.4%)	93 (31.0%)	
Agree	664 (55.0%)	158 (52.7%)	168 (56.0%)	176 (57.3%)	162 (54.0%)	
Strongly agree	169 (14.0%)	63 (21.0%)	43 (14.3%)	32 (10.4%)	31 (10.3%)	
The CDC believes in what they recommend for the public						**<0.01**
Strongly disagree	40 (3.3%)	3 (1.0%)	7 (2.3%)	16 (5.2%)	14 (4.7%)	
Disagree	144 (11.9%)	24 (8.0%)	35 (11.7%)	46 (15.0%)	39 (13.0%)	
Agree	739 (61.2%)	171 (57.0%)	182 (60.7%)	191 (62.2%)	195 (65.0%)	
Strongly agree	284 (23.5%)	102 (34.0%)	76 (25.3%)	54 (17.6%)	52 (17.3%)	
The CDC recommends things for the public that are not helpful [c]						**<0.01**
Strongly agree	102 (8.5%)	18 (6.0%)	35 (11.7%)	26 (8.5%)	23 (7.7%)	
Agree	367 (30.4%)	73 (24.3%)	93 (31.0%)	102 (33.2%)	99 (33.0%)	
Disagree	601 (49.8%)	150 (50.0%)	141 (47.0%)	161 (52.4%)	149 (49.7%)	
Strongly disagree	137 (11.4%)	59 (19.7%)	31 (10.3%)	18 (5.9%)	29 (9.7%)	

[a] PA = Physician Assistant; NP = Nurse Practitioner

[b] boldface indicates statistical significance (p<0.05) using Pearson's Chi-Squared Test

[c] Responses to 4-point Likert scale items used as the basis for the composite scale centralized around the middle options of "agree" and disagree" compared to "strongly agree" and "strongly disagree." Response options were scored and summed to create linear scores: strongly agree=3, agree=2, disagree=1, strongly disagree=0. Selected items ([c]) were reversed: strongly agree=0, agree=1, disagree=2, strongly disagree=3. These scores were then divided by the maximum to create a scale with possible scores from 0 to 100 (e.g., 100 being complete trust and 0 being complete distrust). These continuous scores were also dichotomized at the middle (50) to allow for logistic analyses.

[d] Cronbach's alpha is a measure of internal consistency. Scales with Cronbach alpha values greater than 0.80 are generally considered to have satisfactory reliability.

Supplementary Table 2. ***Characteristics of Participating Healthcare Personnel, Their Practices, and Their Patient Populations, by Healthcare Personnel Booster Status***

	Total N=1,207	Not Boosted N=218	Boosted N=989	p-value[b]
PRACTICE CHARACTERISTICS				
Practice location, urban/suburban/rural				**0.03**
Urban	446 (37.0%)	77 (35.3%)	369 (37.3%)	
Suburban	590 (48.9%)	98 (45.0%)	492 (49.7%)	
Rural	171 (14.2%)	43 (19.7%)	128 (12.9%)	
U.S. region, assigned				**<0.01**
Northeast	278 (23.0%)	43 (19.7%)	235 (23.8%)	
Midwest	325 (26.9%)	57 (26.1%)	268 (27.1%)	
South	402 (33.3%)	96 (44.0%)	306 (30.9%)	
West	202 (16.7%)	22 (10.1%)	180 (18.2%)	
Practice setting				0.76
Private, independent practice	498 (41.3%)	77 (35.3%)	421 (42.6%)	
Practice network/HMO	105 (8.7%)	18 (8.3%)	87 (8.8%)	
Hospital or medical center	227 (18.8%)	37 (17.0%)	190 (19.2%)	
Community health center/Federally Qualified Health Center (FQHC)	59 (4.9%)	6 (2.8%)	53 (5.4%)	
Other	18 (1.5%)	2 (0.9%)	16 (1.6%)	
Missing	300 (24.9%)	78 (35.8%)	222 (22.4%)	
Average number of patients per day				**0.02**
<10	44 (3.6%)	10 (4.6%)	34 (3.4%)	
10-24	613 (50.8%)	92 (42.2%)	521 (52.7%)	
>=25	550 (45.6%)	116 (53.2%)	434 (43.9%)	
Service population				**<0.01**
Children (<18 yrs)	193 (16.0%)	17 (7.8%)	176 (17.8%)	
Adults (>=18 yrs)	199 (16.5%)	41 (18.8%)	158 (16.0%)	
Both children and adults	815 (67.5%)	160 (73.4%)	655 (66.2%)	
Practice currently administers vaccines	1,168 (96.8%)	210 (96.3%)	958 (96.9%)	0.69
Practice provided seasonal influenza vaccination: 2019-2020	597 (92.1%)	105 (86.1%)	492 (93.5%)	**<0.01**
Practice provided seasonal influenza vaccination: 2020-2021	609 (94.0%)	111 (91.0%)	498 (94.7%)	0.12
Practice provided seasonal influenza vaccination: 2021-2022	607 (93.7%)	107 (87.7%)	500 (95.1%)	**<0.01**
Practice provides COVID-19 vaccines	881 (73.0%)	157 (72.0%)	724 (73.2%)	0.72
Pfizer COVID-19 vax	758 (86.0%)	129 (82.2%)	629 (86.9%)	0.12
Moderna COVID-19 vax	632 (71.7%)	115 (73.2%)	517 (71.4%)	0.64
J&J COVID-19 vax	107 (12.1%)	35 (22.3%)	72 (9.9%)	**<0.01**
Novavax COVID-19 vax	60 (6.8%)	11 (7.0%)	49 (6.8%)	0.91
Strategies used to improve COVID-19 vaccine series completion:				

paper-based reminder card	409 (46.4%)	83 (52.9%)	326 (45.0%)	0.07
reminder telephone calls	347 (39.4%)	56 (35.7%)	291 (40.2%)	0.29
reminder text messages	299 (33.9%)	57 (36.3%)	242 (33.4%)	0.49
reminder emails	283 (32.1%)	45 (28.7%)	238 (32.9%)	0.31
flagging patient charts	274 (31.1%)	37 (23.6%)	237 (32.7%)	**0.02**
scheduling next dose at current visit	605 (68.7%)	104 (66.2%)	501 (69.2%)	0.47
computerized immunization database/registry	342 (38.8%)	49 (31.2%)	293 (40.5%)	**0.03**
Practice participates in the Vaccines for Children (VFC) program	612 (52.4%)	92 (43.8%)	520 (54.3%)	**<0.01**
Practice uses Electronic Health Records (EHR)	952 (78.9%)	148 (67.9%)	804 (81.3%)	**<0.01**
PATIENT CHARACTERISTICS				
Percent insured, private insurance				**0.03**
<25%	172 (14.3%)	27 (12.4%)	145 (14.7%)	
25-50%	439 (36.4%)	94 (43.1%)	345 (34.9%)	
51-75%	352 (29.2%)	68 (31.2%)	284 (28.7%)	
>75%	229 (19.0%)	27 (12.4%)	202 (20.4%)	
Unsure	15 (1.2%)	2 (0.9%)	13 (1.3%)	
Percent insured, Medicaid/CHIP				0.17
<25%	608 (50.4%)	98 (45.0%)	510 (51.6%)	
25-50%	411 (34.1%)	90 (41.3%)	321 (32.5%)	
51-75%	108 (8.9%)	18 (8.3%)	90 (9.1%)	
>75%	44 (3.6%)	6 (2.8%)	38 (3.8%)	
Unsure	36 (3.0%)	6 (2.8%)	30 (3.0%)	
Percent insured, Medicare				**<0.01**
<25%	470 (38.9%)	62 (28.4%)	408 (41.3%)	
25-50%	496 (41.1%)	116 (53.2%)	380 (38.4%)	
51-75%	130 (10.8%)	23 (10.6%)	107 (10.8%)	
>75%	44 (3.6%)	13 (6.0%)	31 (3.1%)	
Unsure	67 (5.6%)	4 (1.8%)	63 (6.4%)	
Percent insured, Uninsured				0.05
<25%	1,058 (87.7%)	196 (89.9%)	862 (87.2%)	
25-50%	51 (4.2%)	13 (6.0%)	38 (3.8%)	
51-75%	7 (0.6%)	0 (0.0%)	7 (0.7%)	
>75%	7 (0.6%)	2 (0.9%)	5 (0.5%)	
Unsure	84 (7.0%)	7 (3.2%)	77 (7.8%)	
Percent race/ethnicity, Hispanic/Latino				0.44
<25%	754 (62.5%)	128 (58.7%)	626 (63.3%)	
25-50%	352 (29.2%)	69 (31.7%)	283 (28.6%)	
51-75%	70 (5.8%)	12 (5.5%)	58 (5.9%)	
>75%	21 (1.7%)	6 (2.8%)	15 (1.5%)	

Unsure	10 (0.8%)	3 (1.4%)	7 (0.7%)	
Percent race/ethnicity, Black/African American				0.45
<25%	711 (58.9%)	118 (54.1%)	593 (60.0%)	
25-50%	399 (33.1%)	78 (35.8%)	321 (32.5%)	
51-75%	66 (5.5%)	14 (6.4%)	52 (5.3%)	
>75%	21 (1.7%)	6 (2.8%)	15 (1.5%)	
Unsure	10 (0.8%)	2 (0.9%)	8 (0.8%)	
Percent race/ethnicity, Asian				0.05
<25%	1,035 (85.7%)	194 (89.0%)	841 (85.0%)	
25-50%	142 (11.8%)	18 (8.3%)	124 (12.5%)	
51-75%	12 (1.0%)	0 (0.0%)	12 (1.2%)	
>75%	7 (0.6%)	3 (1.4%)	4 (0.4%)	
Unsure	11 (0.9%)	3 (1.4%)	8 (0.8%)	
Percent race/ethnicity, Other minority group				0.14
<25%	202 (16.7%)	33 (15.1%)	169 (17.1%)	
25-50%	24 (2.0%)	8 (3.7%)	16 (1.6%)	
51-75%	15 (1.2%)	4 (1.8%)	11 (1.1%)	
>75%	9 (0.7%)	3 (1.4%)	6 (0.6%)	
Unsure	117 (9.7%)	29 (13.3%)	88 (8.9%)	
Missing	840 (69.6%)	141 (64.7%)	699 (70.7%)	
PROVIDER CHARACTERISTICS				
Current medical profession				**<0.01**
Physician	600 (49.7%)	58 (26.6%)	542 (54.8%)	
Physician Assistant	100 (8.3%)	26 (11.9%)	74 (7.5%)	
Nurse Practitioner	105 (8.7%)	27 (12.4%)	78 (7.9%)	
Nurse (RN or LPN)	102 (8.5%)	29 (13.3%)	73 (7.4%)	
Pharmacist	300 (24.9%)	78 (35.8%)	222 (22.4%)	
Specialty				**<0.01**
Internal Medicine	118 (9.8%)	34 (15.6%)	84 (8.5%)	
Family Practice	458 (37.9%)	81 (37.2%)	377 (38.1%)	
General Pediatrics	331 (27.4%)	25 (11.5%)	306 (30.9%)	
Missing	300 (24.9%)	78 (35.8%)	222 (22.4%)	
Highest clinical degree				**<0.01**
Associate degree	22 (1.8%)	5 (2.3%)	17 (1.7%)	
Bachelor's degree	162 (13.4%)	45 (20.6%)	117 (11.8%)	
Master's degree	206 (17.1%)	51 (23.4%)	155 (15.7%)	
Doctorate level	811 (67.2%)	114 (52.3%)	697 (70.5%)	
Missing	6 (0.5%)	3 (1.4%)	3 (0.3%)	
Graduation year, highest completed clinical degree				**<0.01**

<1980	52 (4.3%)	0 (0.0%)	52 (5.3%)	
1980-1989	195 (16.2%)	24 (11.0%)	171 (17.3%)	
1990-1999	318 (26.3%)	52 (23.9%)	266 (26.9%)	
2000-2009	380 (31.5%)	80 (36.7%)	300 (30.3%)	
2010-2021	247 (20.5%)	57 (26.1%)	190 (19.2%)	
Missing	15 (1.2%)	5 (2.3%)	10 (1.0%)	
Race/Ethnicity				**<0.01**
White	832 (68.9%)	146 (67.0%)	686 (69.4%)	
Asian	158 (13.1%)	16 (7.3%)	142 (14.4%)	
Black	48 (4.0%)	14 (6.4%)	34 (3.4%)	
Hispanic	37 (3.1%)	9 (4.1%)	28 (2.8%)	
Other	38 (3.1%)	11 (5.0%)	27 (2.7%)	
Missing	94 (7.8%)	22 (10.1%)	72 (7.3%)	
Regularly taken care of COVID-19 patients	1,069 (88.6%)	192 (88.1%)	877 (88.7%)	0.80
Received at least one COVID-19 vaccine	1,154 (95.6%)	165 (75.7%)	989 (100.0%)	**<0.01**
COVID-19 vaccination should be _____ for healthcare workers				**<0.01**
Voluntary	464 (38.4%)	167 (76.6%)	297 (30.0%)	
Mandated	560 (46.4%)	14 (6.4%)	546 (55.2%)	
Not sure	183 (15.2%)	37 (17.0%)	146 (14.8%)	
High Trust in CDC [c]	883 (73.2%)	92 (42.2%)	791 (80.0%)	**<0.01**

[a] PA = Physician Assistant; NP = Nurse Practitioner

[b] boldface indicates statistical significance (p<0.05) using Pearson's Chi-Squared Test

[c] CDC = Centers for Disease Control and Prevention; see Supplementary Table 1

Supplementary Table 3. ***Impact of the Pandemic on Routine Vaccination by Healthcare Personnel Booster Status***

	Total N=1,207	Not Boosted N=218	Boosted N=989	p-value[b]
Included telehealth visits before March 2020	228 (18.9%)	40 (18.3%)	188 (19.0%)	0.82
% of total visits telehealth before March 2020				0.44
0-24%	204 (89.5%)	35 (87.5%)	169 (89.9%)	
25-49%	9 (3.9%)	3 (7.5%)	6 (3.2%)	
50-74%	10 (4.4%)	2 (5.0%)	8 (4.3%)	
75-100%	5 (2.2%)	0 (0.0%)	5 (2.7%)	
Included telehealth visits since March 2020	971 (80.4%)	163 (74.8%)	808 (81.7%)	**0.02**
% of total visits telehealth before March 2020				0.78
0-24%	627 (64.6%)	104 (63.8%)	523 (64.7%)	
25-49%	256 (26.4%)	46 (28.2%)	210 (26.0%)	
50-74%	75 (7.7%)	12 (7.4%)	63 (7.8%)	
75-100%	13 (1.3%)	1 (0.6%)	12 (1.5%)	
Plan to continue telehealth after pandemic	846 (87.1%)	140 (85.9%)	706 (87.4%)	0.61
Decreased ability to vaccinate due to telehealth	487 (50.2%)	90 (55.2%)	397 (49.1%)	0.16
Obstacles to vaccinating during pandemic so far:				
decreased access to patients	790 (65.5%)	122 (56.0%)	668 (67.5%)	**<0.01**
lenient enforcement of school requirements	342 (28.3%)	62 (28.4%)	280 (28.3%)	0.97
disruption of vaccine supply	459 (38.0%)	95 (43.6%)	364 (36.8%)	0.06
staffing and PPE shortages	442 (36.6%)	88 (40.4%)	354 (35.8%)	0.20
Obstacles to vaccinating expected in the future:				
decreased access to patients	540 (68.4%)	81 (66.4%)	459 (68.7%)	0.61
lenient enforcement of school requirements	231 (67.5%)	42 (67.7%)	189 (67.5%)	0.97
disruption of vaccine supply	324 (70.6%)	65 (68.4%)	259 (71.2%)	0.60
staffing and PPE shortages	302 (68.3%)	58 (65.9%)	244 (68.9%)	0.59
More patients concerned about routine vaccines since pandemic	862 (71.4%)	165 (75.7%)	697 (70.5%)	0.12
More patients refusing routine vaccines since pandemic	685 (56.8%)	124 (56.9%)	561 (56.7%)	0.97
Since March 2020, practice implemented changes to boost vaccination	648 (53.7%)	122 (56.0%)	526 (53.2%)	0.46
patient-focused	530 (81.8%)	99 (81.1%)	431 (81.9%)	0.84
provider-focused	378 (58.3%)	67 (54.9%)	311 (59.1%)	0.40
practice-focused	381 (58.8%)	63 (51.6%)	318 (60.5%)	0.08
improved vaccine availability and access	347 (53.5%)	65 (53.3%)	282 (53.6%)	0.95
Practice stopped routine vaccines since March 2020	69 (10.9%)	17 (14.5%)	52 (10.1%)	0.17

[a] PA = Physician Assistant; NP = Nurse Practitioner

[b] boldface indicates statistical significance ($p<0.05$) using Pearson's Chi-Squared Test

Supplementary Table 4. ***Vaccine Resources, Discussions, Recommendations, and Barriers, by Healthcare Personnel Booster Status***

	Total N=1,207	Not Boosted N=218	Boosted N=989	p-value[b]
Average hours per week spent talking with patients about vaccines				0.13
0-1	182 (15.1%)	27 (12.4%)	155 (15.7%)	
2-3	439 (36.4%)	69 (31.7%)	370 (37.4%)	
4-5	275 (22.8%)	52 (23.9%)	223 (22.5%)	
6-9	79 (6.5%)	20 (9.2%)	59 (6.0%)	
10-19	151 (12.5%)	30 (13.8%)	121 (12.2%)	
20+	81 (6.7%)	20 (9.2%)	61 (6.2%)	
Proportion of patients with vaccine concerns				**<0.01**
<25%	587 (49.2%)	71 (33.3%)	516 (52.6%)	
25-50%	416 (34.8%)	92 (43.2%)	324 (33.0%)	
51-75%	143 (12.0%)	32 (15.0%)	111 (11.3%)	
76-100%	48 (4.0%)	18 (8.5%)	30 (3.1%)	
Proportion of vaccination visits billed for administration				**0.01**
<25%	180 (17.5%)	40 (22.2%)	140 (16.5%)	
25-50%	123 (11.9%)	31 (17.2%)	92 (10.8%)	
51-75%	80 (7.8%)	13 (7.2%)	67 (7.9%)	
76-100%	647 (62.8%)	96 (53.3%)	551 (64.8%)	
Proportion of vaccination visits billed for counseling				**<0.01**
<25%	691 (68.6%)	128 (69.2%)	563 (68.4%)	
25-50%	93 (9.2%)	27 (14.6%)	66 (8.0%)	
51-75%	69 (6.8%)	12 (6.5%)	57 (6.9%)	
76-100%	155 (15.4%)	18 (9.7%)	137 (16.6%)	
Often used for vaccine information:				
News media	140 (11.6%)	14 (6.4%)	126 (12.7%)	**<0.01**
Social media	74 (6.1%)	15 (6.9%)	59 (6.0%)	0.61
Private social media groups	65 (5.4%)	9 (4.1%)	56 (5.7%)	0.36
Email newsletters/listservs	242 (20.0%)	47 (21.6%)	195 (19.7%)	0.54
Websites	579 (48.0%)	102 (46.8%)	477 (48.2%)	0.70
Blogs	50 (4.1%)	17 (7.8%)	33 (3.3%)	**<0.01**
Message boards	68 (5.6%)	12 (5.5%)	56 (5.7%)	0.93
Text message alerts	81 (6.7%)	14 (6.4%)	67 (6.8%)	0.85
Podcasts	118 (9.8%)	27 (12.4%)	91 (9.2%)	0.15
Publications in academic/medical journals	898 (74.4%)	155 (71.1%)	743 (75.1%)	0.22

Trusted for vaccine information:				
News media	102 (8.5%)	8 (3.7%)	94 (9.5%)	**<0.01**
Social media	41 (3.4%)	9 (4.1%)	32 (3.2%)	0.51
Academic/medical journals	1,052 (87.2%)	151 (69.3%)	901 (91.1%)	**<0.01**
Academic/medical institutions	1,029 (85.3%)	138 (63.3%)	891 (90.1%)	**<0.01**
Professional medical organizations	1,023 (84.8%)	132 (60.6%)	891 (90.1%)	**<0.01**
Other healthcare providers	707 (58.6%)	100 (45.9%)	607 (61.4%)	**<0.01**
Centers for Disease Control and Prevention (CDC)	933 (77.3%)	96 (44.0%)	837 (84.6%)	**<0.01**
Food and Drug Administration (FDA)	886 (73.4%)	99 (45.4%)	787 (79.6%)	**<0.01**
State and local public health departments	870 (72.1%)	93 (42.7%)	777 (78.6%)	**<0.01**
Vaccine-focused non-profit organizations	532 (44.1%)	55 (25.2%)	477 (48.2%)	**<0.01**
Often used to share vaccine information with patients:				
One-on-one conversation	1,032 (85.5%)	174 (79.8%)	858 (86.8%)	**<0.01**
Email newsletter	79 (6.5%)	13 (6.0%)	66 (6.7%)	0.70
My social media accounts	51 (4.2%)	10 (4.6%)	41 (4.1%)	0.77
Other credible social media accounts	57 (4.7%)	15 (6.9%)	42 (4.2%)	0.10
My (or my practice's) website	276 (22.9%)	42 (19.3%)	234 (23.7%)	0.16
Other credible websites	291 (24.1%)	48 (22.0%)	243 (24.6%)	0.43
Videos	98 (8.1%)	23 (10.6%)	75 (7.6%)	0.15
Posters/flyers/brochures in office	413 (34.2%)	67 (30.7%)	346 (35.0%)	0.23
It's easy to stay up-to-date on vaccine recommendations, contraindications, controversies	812 (67.3%)	127 (58.3%)	685 (69.3%)	**<0.01**
Patients sometimes ask vaccine questions to which you are unsure of the scientific answer	810 (67.1%)	148 (67.9%)	662 (66.9%)	0.79
It'd be helpful to know a patient's vaccine intent and concerns prior to a visit	1,040 (86.2%)	175 (80.3%)	865 (87.5%)	**<0.01**
Feel well prepared for vaccine conversations with patients	1,047 (86.7%)	171 (78.4%)	876 (88.6%)	**<0.01**
Have everything needed to share vaccine info with patients	922 (76.4%)	161 (73.9%)	761 (76.9%)	0.33
More information would help me recommend COVID-19 vaccines to my patients	192 (21.8%)	35 (22.3%)	157 (21.7%)	0.87
Interest in a CME module on how to discuss COVID-19 and other vaccines with patients	472 (39.1%)	72 (33.0%)	400 (40.4%)	**0.04**
Interest in online resource for HCP detailing how to talk with patients, vaccine recommendations, and vaccine safety issues	792 (65.6%)	120 (55.0%)	672 (67.9%)	**<0.01**
Interest in website to refer patients to that provides them regularly updated and individually tailored vaccine info	798 (66.1%)	120 (55.0%)	678 (68.6%)	**<0.01**
Adverse Event Reporting [c]				
Familiar with VAERS	566 (93.7%)	102 (94.4%)	464 (93.5%)	0.73
Familiar with (fictitious) IARM system	475 (78.8%)	89 (80.9%)	386 (78.3%)	0.54
Ever reported to VAERS	195 (34.5%)	34 (33.3%)	161 (34.7%)	0.79

Ever reported to (fictitious) IARM system	120 (25.3%)	25 (28.1%)	95 (24.6%)	0.50
COVID-19 vaccine recommendations by vaccine				
Pfizer	403 (33.4%)	26 (11.9%)	377 (38.1%)	**<0.01**
Moderna	480 (39.8%)	25 (11.5%)	455 (46.0%)	**<0.01**
Johnson & Johnson	85 (7.0%)	5 (2.3%)	80 (8.1%)	**<0.01**
Novavax	107 (8.9%)	14 (6.4%)	93 (9.4%)	0.16
COVID-19 vaccine recommendations for specific patient populations				
high-risk patients	790 (65.5%)	68 (31.2%)	722 (73.0%)	**<0.01**
patients that live with or care for high-risk persons	803 (66.5%)	62 (28.4%)	741 (74.9%)	**<0.01**
6-23-month-old patients	265 (29.0%)	5 (2.9%)	260 (35.0%)	**<0.01**
2-4-year-old patients	311 (32.3%)	11 (6.1%)	300 (38.4%)	**<0.01**
5-11-year-old patients	431 (41.9%)	17 (8.9%)	414 (49.5%)	**<0.01**
12-15-year-old patients	499 (47.3%)	22 (11.4%)	477 (55.3%)	**<0.01**
16-24-year-old patients	607 (51.7%)	30 (14.2%)	577 (59.9%)	**<0.01**
25-64-year-old patients	593 (61.6%)	43 (21.3%)	550 (72.3%)	**<0.01**
65+ year-old patients	748 (78.7%)	87 (42.2%)	661 (88.7%)	**<0.01**
Routine vaccine recommendations for eligible patients by vaccine				
influenza	853 (70.7%)	80 (36.7%)	773 (78.2%)	**<0.01**
routine childhood (e.g., MMR, DTaP)	887 (82.9%)	121 (65.4%)	766 (86.6%)	**<0.01**
HPV	674 (59.3%)	76 (36.7%)	598 (64.4%)	**<0.01**
shingles	644 (66.2%)	94 (46.3%)	550 (71.4%)	**<0.01**
pneumococcal	944 (79.6%)	127 (58.3%)	817 (84.4%)	**<0.01**
Obstacles to administering COVID-19 vaccines:				
believe not needed for some patients	177 (14.7%)	50 (22.9%)	127 (12.8%)	**<0.01**
time it takes to discuss with patients	180 (14.9%)	33 (15.1%)	147 (14.9%)	0.92
uncertainty amount adequate reimbursement	156 (12.9%)	25 (11.5%)	131 (13.2%)	0.48
general administrative burden	370 (30.7%)	56 (25.7%)	314 (31.7%)	0.08
additional workload of another vaccine	325 (26.9%)	68 (31.2%)	257 (26.0%)	0.12
patient concerns about COVID-19 vaccine safety	729 (60.4%)	138 (63.3%)	591 (59.8%)	0.33
patient concerns about general vaccine safety	575 (47.6%)	115 (52.8%)	460 (46.5%)	0.10
patient concerns about COVID-19 vaccine necessity	776 (64.3%)	126 (57.8%)	650 (65.7%)	**0.03**
patient concerns about COVID-19 vaccine effectiveness	654 (54.2%)	120 (55.0%)	534 (54.0%)	0.78

[a] PA = Physician Assistant; NP = Nurse Practitioner

[b] boldface indicates statistical significance (p<0.05) using Pearson's Chi-Squared Test

[c] VAERS = Vaccine Adverse Event Reporting System; IARM = Immunization Adverse Reaction Monitoring (fictitious)

Supplementary Table 5. ***Characteristics of Participating Healthcare Personnel, Their Practices, and Their Patient Populations, by Practice Urbanicity***

	Total N=1,207	Urban N=446	Suburban N=590	Rural N=171	p-value[b]
PRACTICE CHARACTERISTICS					
U.S. region, assigned					**<0.01**
Northeast	278 (23.0%)	99 (22.2%)	153 (25.9%)	26 (15.2%)	
Midwest	325 (26.9%)	113 (25.3%)	154 (26.1%)	58 (33.9%)	
South	402 (33.3%)	143 (32.1%)	196 (33.2%)	63 (36.8%)	
West	202 (16.7%)	91 (20.4%)	87 (14.7%)	24 (14.0%)	
Practice setting					**<0.01**
Private, independent practice	498 (41.3%)	137 (30.7%)	299 (50.7%)	62 (36.3%)	
Practice network/HMO	105 (8.7%)	29 (6.5%)	61 (10.3%)	15 (8.8%)	
Hospital or medical center	227 (18.8%)	130 (29.1%)	73 (12.4%)	24 (14.0%)	
Community health center/Federally Qualified Health Center (FQHC)	59 (4.9%)	26 (5.8%)	13 (2.2%)	20 (11.7%)	
Other	18 (1.5%)	7 (1.6%)	7 (1.2%)	4 (2.3%)	
Missing	300 (24.9%)	117 (26.2%)	137 (23.2%)	46 (26.9%)	
Average number of patients per day					0.10
<10	44 (3.6%)	23 (5.2%)	14 (2.4%)	7 (4.1%)	
10-24	613 (50.8%)	229 (51.3%)	292 (49.5%)	92 (53.8%)	
>=25	550 (45.6%)	194 (43.5%)	284 (48.1%)	72 (42.1%)	
Service population					**0.03**
Children (<18 yrs)	193 (16.0%)	74 (16.6%)	103 (17.5%)	16 (9.4%)	
Adults (>=18 yrs)	199 (16.5%)	82 (18.4%)	95 (16.1%)	22 (12.9%)	
Both children and adults	815 (67.5%)	290 (65.0%)	392 (66.4%)	133 (77.8%)	
Practice currently administers vaccines	1,168 (96.8%)	430 (96.4%)	574 (97.3%)	164 (95.9%)	0.58
Practice provided seasonal influenza vaccination: 2019-2020	597 (92.1%)	242 (93.1%)	274 (91.3%)	81 (92.0%)	0.75
Practice provided seasonal influenza vaccination: 2020-2021	609 (94.0%)	247 (95.0%)	278 (92.7%)	84 (95.5%)	0.42
Practice provided seasonal influenza vaccination: 2021-2022	607 (93.7%)	243 (93.5%)	281 (93.7%)	83 (94.3%)	0.96
Practice provides COVID-19 vaccines	881 (73.0%)	347 (77.8%)	408 (69.2%)	126 (73.7%)	**<0.01**
Pfizer COVID-19 vax	758 (86.0%)	307 (88.5%)	354 (86.8%)	97 (77.0%)	**<0.01**
Moderna COVID-19 vax	632 (71.7%)	252 (72.6%)	279 (68.4%)	101 (80.2%)	**0.03**
J&J COVID-19 vax	107 (12.1%)	47 (13.5%)	47 (11.5%)	13 (10.3%)	0.55
Novavax COVID-19 vax	60 (6.8%)	24 (6.9%)	31 (7.6%)	5 (4.0%)	0.37
Strategies used to improve COVID-19 vaccine series completion:					
paper-based reminder card	409 (46.4%)	177 (51.0%)	172 (42.2%)	60 (47.6%)	0.05
reminder telephone calls	347 (39.4%)	139 (40.1%)	155 (38.0%)	53 (42.1%)	0.68
reminder text messages	299 (33.9%)	125 (36.0%)	130 (31.9%)	44 (34.9%)	0.47
reminder emails	283 (32.1%)	124 (35.7%)	129 (31.6%)	30 (23.8%)	**0.05**

flagging patient charts	274 (31.1%)	106 (30.5%)	129 (31.6%)	39 (31.0%)	0.95
scheduling next dose at current visit	605 (68.7%)	234 (67.4%)	287 (70.3%)	84 (66.7%)	0.60
computerized immunization database/registry	342 (38.8%)	128 (36.9%)	161 (39.5%)	53 (42.1%)	0.56
Practice participates in the Vaccines for Children (VFC) program	612 (52.4%)	231 (53.7%)	282 (49.1%)	99 (60.4%)	0.03
Practice uses Electronic Health Records (EHR)	952 (78.9%)	352 (78.9%)	462 (78.3%)	138 (80.7%)	0.80
PATIENT CHARACTERISTICS					
Percent insured, private insurance					**<0.01**
<25%	172 (14.3%)	83 (18.6%)	44 (7.5%)	45 (26.3%)	
25-50%	439 (36.4%)	167 (37.4%)	187 (31.7%)	85 (49.7%)	
51-75%	352 (29.2%)	119 (26.7%)	203 (34.4%)	30 (17.5%)	
>75%	229 (19.0%)	69 (15.5%)	150 (25.4%)	10 (5.8%)	
Unsure	15 (1.2%)	8 (1.8%)	6 (1.0%)	1 (0.6%)	
Percent insured, Medicaid/CHIP					**<0.01**
<25%	608 (50.4%)	190 (42.6%)	359 (60.8%)	59 (34.5%)	
25-50%	411 (34.1%)	154 (34.5%)	176 (29.8%)	81 (47.4%)	
51-75%	108 (8.9%)	57 (12.8%)	31 (5.3%)	20 (11.7%)	
>75%	44 (3.6%)	30 (6.7%)	6 (1.0%)	8 (4.7%)	
Unsure	36 (3.0%)	15 (3.4%)	18 (3.1%)	3 (1.8%)	
Percent insured, Medicare					**<0.01**
<25%	470 (38.9%)	182 (40.8%)	252 (42.7%)	36 (21.1%)	
25-50%	496 (41.1%)	167 (37.4%)	228 (38.6%)	101 (59.1%)	
51-75%	130 (10.8%)	45 (10.1%)	64 (10.8%)	21 (12.3%)	
>75%	44 (3.6%)	15 (3.4%)	21 (3.6%)	8 (4.7%)	
Unsure	67 (5.6%)	37 (8.3%)	25 (4.2%)	5 (2.9%)	
Percent insured, Uninsured					**<0.01**
<25%	1,058 (87.7%)	367 (82.3%)	539 (91.4%)	152 (88.9%)	
25-50%	51 (4.2%)	26 (5.8%)	15 (2.5%)	10 (5.8%)	
51-75%	7 (0.6%)	4 (0.9%)	2 (0.3%)	1 (0.6%)	
>75%	7 (0.6%)	4 (0.9%)	2 (0.3%)	1 (0.6%)	
Unsure	84 (7.0%)	45 (10.1%)	32 (5.4%)	7 (4.1%)	
Percent race/ethnicity, Hispanic/Latino					**<0.01**
<25%	754 (62.5%)	213 (47.8%)	399 (67.6%)	142 (83.0%)	
25-50%	352 (29.2%)	177 (39.7%)	153 (25.9%)	22 (12.9%)	
51-75%	70 (5.8%)	38 (8.5%)	27 (4.6%)	5 (2.9%)	
>75%	21 (1.7%)	13 (2.9%)	6 (1.0%)	2 (1.2%)	
Unsure	10 (0.8%)	5 (1.1%)	5 (0.8%)	0 (0.0%)	
Percent race/ethnicity, Black/African American					**<0.01**
<25%	711 (58.9%)	210 (47.1%)	371 (62.9%)	130 (76.0%)	
25-50%	399 (33.1%)	177 (39.7%)	189 (32.0%)	33 (19.3%)	

51-75%	66 (5.5%)	38 (8.5%)	21 (3.6%)	7 (4.1%)	
>75%	21 (1.7%)	17 (3.8%)	3 (0.5%)	1 (0.6%)	
Unsure	10 (0.8%)	4 (0.9%)	6 (1.0%)	0 (0.0%)	
Percent race/ethnicity, Asian					**<0.01**
<25%	1,035 (85.7%)	350 (78.5%)	519 (88.0%)	166 (97.1%)	
25-50%	142 (11.8%)	78 (17.5%)	61 (10.3%)	3 (1.8%)	
51-75%	12 (1.0%)	6 (1.3%)	6 (1.0%)	0 (0.0%)	
>75%	7 (0.6%)	7 (1.6%)	0 (0.0%)	0 (0.0%)	
Unsure	11 (0.9%)	5 (1.1%)	4 (0.7%)	2 (1.2%)	
Percent race/ethnicity, Other minority group					0.09
<25%	202 (16.7%)	69 (15.5%)	103 (17.5%)	30 (17.5%)	
25-50%	24 (2.0%)	12 (2.7%)	8 (1.4%)	4 (2.3%)	
51-75%	15 (1.2%)	3 (0.7%)	8 (1.4%)	4 (2.3%)	
>75%	9 (0.7%)	2 (0.4%)	3 (0.5%)	4 (2.3%)	
Unsure	117 (9.7%)	46 (10.3%)	59 (10.0%)	12 (7.0%)	
Missing	840 (69.6%)	314 (70.4%)	409 (69.3%)	117 (68.4%)	
<u>PROVIDER CHARACTERISTICS</u>					
Current medical profession					**0.04**
Physician	600 (49.7%)	207 (46.4%)	321 (54.4%)	72 (42.1%)	
Physician Assistant	100 (8.3%)	34 (7.6%)	45 (7.6%)	21 (12.3%)	
Nurse Practitioner	105 (8.7%)	41 (9.2%)	48 (8.1%)	16 (9.4%)	
Nurse (RN or LPN)	102 (8.5%)	47 (10.5%)	39 (6.6%)	16 (9.4%)	
Pharmacist	300 (24.9%)	117 (26.2%)	137 (23.2%)	46 (26.9%)	
Specialty					**<0.01**
Internal Medicine	118 (9.8%)	55 (12.3%)	50 (8.5%)	13 (7.6%)	
Family Practice	458 (37.9%)	146 (32.7%)	224 (38.0%)	88 (51.5%)	
General Pediatrics	331 (27.4%)	128 (28.7%)	179 (30.3%)	24 (14.0%)	
Missing	300 (24.9%)	117 (26.2%)	137 (23.2%)	46 (26.9%)	
Highest clinical degree					0.23
Associate degree	22 (1.8%)	6 (1.3%)	11 (1.9%)	5 (2.9%)	
Bachelor's degree	162 (13.4%)	66 (14.8%)	69 (11.7%)	27 (15.8%)	
Master's degree	206 (17.1%)	83 (18.6%)	91 (15.4%)	32 (18.7%)	
Doctorate level	811 (67.2%)	290 (65.0%)	415 (70.3%)	106 (62.0%)	
Missing	6 (0.5%)	1 (0.2%)	4 (0.7%)	1 (0.6%)	
Graduation year, highest completed clinical degree					**<0.01**
<1980	52 (4.3%)	11 (2.5%)	32 (5.4%)	9 (5.3%)	
1980-1989	195 (16.2%)	55 (12.3%)	108 (18.3%)	32 (18.7%)	
1990-1999	318 (26.3%)	100 (22.4%)	173 (29.3%)	45 (26.3%)	
2000-2009	380 (31.5%)	151 (33.9%)	175 (29.7%)	54 (31.6%)	

2010-2021	247 (20.5%)	119 (26.7%)	100 (16.9%)	28 (16.4%)	
Missing	15 (1.2%)	10 (2.2%)	2 (0.3%)	3 (1.8%)	
Race/Ethnicity					**<0.01**
White	832 (68.9%)	253 (56.7%)	443 (75.1%)	136 (79.5%)	
Asian	158 (13.1%)	86 (19.3%)	63 (10.7%)	9 (5.3%)	
Black	48 (4.0%)	28 (6.3%)	14 (2.4%)	6 (3.5%)	
Hispanic	37 (3.1%)	13 (2.9%)	19 (3.2%)	5 (2.9%)	
Other	38 (3.1%)	20 (4.5%)	14 (2.4%)	4 (2.3%)	
Missing	94 (7.8%)	46 (10.3%)	37 (6.3%)	11 (6.4%)	
Regularly taken care of COVID-19 patients	1,069 (88.6%)	389 (87.2%)	525 (89.0%)	155 (90.6%)	0.44
Received at least one COVID-19 vaccine	1,154 (95.6%)	431 (96.6%)	566 (95.9%)	157 (91.8%)	**0.03**
Received at least one COVID-19 booster dose	989 (81.9%)	369 (82.7%)	492 (83.4%)	128 (74.9%)	**0.03**
COVID-19 vaccination should be _____ for healthcare workers					**<0.01**
Voluntary	464 (38.4%)	141 (31.6%)	236 (40.0%)	87 (50.9%)	
Mandated	560 (46.4%)	230 (51.6%)	264 (44.7%)	66 (38.6%)	
Not sure	183 (15.2%)	75 (16.8%)	90 (15.3%)	18 (10.5%)	
High Trust in CDC [c]	883 (73.2%)	345 (77.4%)	424 (71.9%)	114 (66.7%)	**0.02**

[a] PA = Physician Assistant; NP = Nurse Practitioner

[b] boldface indicates statistical significance (p<0.05) using Pearson's Chi-Squared Test

[c] CDC = Centers for Disease Control and Prevention; see Supplementary Table 1

Supplementary Table 6. ***Impact of the Pandemic on Routine Vaccination by Practice Urbanicity***

	Total N=1,207	Urban N=446	Suburban N=590	Rural N=171	p-value[b]
Included telehealth visits before March 2020	228 (18.9%)	96 (21.5%)	100 (16.9%)	32 (18.7%)	0.18
% of total visits telehealth before March 2020					0.93
0-24%	204 (89.5%)	86 (89.6%)	89 (89.0%)	29 (90.6%)	
25-49%	9 (3.9%)	3 (3.1%)	4 (4.0%)	2 (6.3%)	
50-74%	10 (4.4%)	4 (4.2%)	5 (5.0%)	1 (3.1%)	
75-100%	5 (2.2%)	3 (3.1%)	2 (2.0%)	0 (0.0%)	
Included telehealth visits since March 2020	971 (80.4%)	367 (82.3%)	478 (81.0%)	126 (73.7%)	**0.05**
% of total visits telehealth before March 2020					**<0.01**
0-24%	627 (64.6%)	210 (57.2%)	330 (69.0%)	87 (69.0%)	
25-49%	256 (26.4%)	111 (30.2%)	113 (23.6%)	32 (25.4%)	
50-74%	75 (7.7%)	38 (10.4%)	30 (6.3%)	7 (5.6%)	
75-100%	13 (1.3%)	8 (2.2%)	5 (1.0%)	0 (0.0%)	
Plan to continue telehealth after pandemic	846 (87.1%)	329 (89.6%)	410 (85.8%)	107 (84.9%)	0.18
Decreased ability to vaccinate due to telehealth	487 (50.2%)	187 (51.0%)	247 (51.7%)	53 (42.1%)	0.15
Obstacles to vaccinating during pandemic so far:					
decreased access to patients	790 (65.5%)	293 (65.7%)	389 (65.9%)	108 (63.2%)	0.79
lenient enforcement of school requirements	342 (28.3%)	133 (29.8%)	169 (28.6%)	40 (23.4%)	0.28
disruption of vaccine supply	459 (38.0%)	176 (39.5%)	213 (36.1%)	70 (40.9%)	0.38
staffing and PPE shortages	442 (36.6%)	167 (37.4%)	212 (35.9%)	63 (36.8%)	0.88
Obstacles to vaccinating expected in the future:					
decreased access to patients	540 (68.4%)	205 (70.0%)	261 (67.1%)	74 (68.5%)	0.73
lenient enforcement of school requirements	231 (67.5%)	89 (66.9%)	115 (68.0%)	27 (67.5%)	0.98
disruption of vaccine supply	324 (70.6%)	121 (68.8%)	150 (70.4%)	53 (75.7%)	0.56
staffing and PPE shortages	302 (68.3%)	117 (70.1%)	143 (67.5%)	42 (66.7%)	0.82
More patients concerned about routine vaccines since pandemic	862 (71.4%)	299 (67.0%)	426 (72.2%)	137 (80.1%)	**<0.01**
More patients refusing routine vaccines since pandemic	685 (56.8%)	249 (55.8%)	326 (55.3%)	110 (64.3%)	0.10
Since March 2020, practice implemented changes to boost vaccination	648 (53.7%)	260 (58.3%)	300 (50.8%)	88 (51.5%)	**0.05**
patient-focused	530 (81.8%)	211 (81.2%)	246 (82.0%)	73 (83.0%)	0.92
provider-focused	378 (58.3%)	152 (58.5%)	172 (57.3%)	54 (61.4%)	0.80
practice-focused	381 (58.8%)	151 (58.1%)	182 (60.7%)	48 (54.5%)	0.56
improved vaccine availability and access	347 (53.5%)	135 (51.9%)	161 (53.7%)	51 (58.0%)	0.62
Practice stopped routine vaccines since March 2020	69 (10.9%)	23 (9.1%)	33 (11.4%)	13 (14.8%)	0.32

[a] PA = Physician Assistant; NP = Nurse Practitioner

[b] boldface indicates statistical significance ($p<0.05$) using Pearson's Chi-Squared Test

Supplementary Table 7. ***Vaccine Resources, Discussions, Recommendations, and Barriers, by Practice Urbanicity***

	Total N=1,207	Urban N=446	Suburban N=590	Rural N=171	p-value[b]
Average hours per week spent talking with patients about vaccines					0.18
0-1	182 (15.1%)	68 (15.2%)	82 (13.9%)	32 (18.7%)	
2-3	439 (36.4%)	152 (34.1%)	225 (38.1%)	62 (36.3%)	
4-5	275 (22.8%)	93 (20.9%)	139 (23.6%)	43 (25.1%)	
6-9	79 (6.5%)	27 (6.1%)	42 (7.1%)	10 (5.8%)	
10-19	151 (12.5%)	67 (15.0%)	68 (11.5%)	16 (9.4%)	
20+	81 (6.7%)	39 (8.7%)	34 (5.8%)	8 (4.7%)	
Proportion of patients with vaccine concerns					0.06
<25%	587 (49.2%)	223 (50.9%)	290 (49.3%)	74 (44.0%)	
25-50%	416 (34.8%)	150 (34.2%)	203 (34.5%)	63 (37.5%)	
51-75%	143 (12.0%)	44 (10.0%)	80 (13.6%)	19 (11.3%)	
76-100%	48 (4.0%)	21 (4.8%)	15 (2.6%)	12 (7.1%)	
Proportion of vaccination visits billed for administration					**0.01**
<25%	180 (17.5%)	77 (21.3%)	78 (15.1%)	25 (16.6%)	
25-50%	123 (11.9%)	50 (13.9%)	58 (11.2%)	15 (9.9%)	
51-75%	80 (7.8%)	35 (9.7%)	31 (6.0%)	14 (9.3%)	
76-100%	647 (62.8%)	199 (55.1%)	351 (67.8%)	97 (64.2%)	
Proportion of vaccination visits billed for counseling					**0.01**
<25%	691 (68.6%)	237 (68.5%)	333 (66.1%)	121 (76.6%)	
25-50%	93 (9.2%)	37 (10.7%)	42 (8.3%)	14 (8.9%)	
51-75%	69 (6.8%)	30 (8.7%)	35 (6.9%)	4 (2.5%)	
76-100%	155 (15.4%)	42 (12.1%)	94 (18.7%)	19 (12.0%)	
Often used for vaccine information:					
News media	140 (11.6%)	68 (15.2%)	63 (10.7%)	9 (5.3%)	**<0.01**
Social media	74 (6.1%)	33 (7.4%)	35 (5.9%)	6 (3.5%)	0.19
Private social media groups	65 (5.4%)	33 (7.4%)	27 (4.6%)	5 (2.9%)	**0.04**
Email newsletters/listservs	242 (20.0%)	111 (24.9%)	106 (18.0%)	25 (14.6%)	**<0.01**
Websites	579 (48.0%)	226 (50.7%)	281 (47.6%)	72 (42.1%)	0.16
Blogs	50 (4.1%)	26 (5.8%)	19 (3.2%)	5 (2.9%)	0.08
Message boards	68 (5.6%)	35 (7.8%)	25 (4.2%)	8 (4.7%)	**0.04**
Text message alerts	81 (6.7%)	35 (7.8%)	38 (6.4%)	8 (4.7%)	0.35
Podcasts	118 (9.8%)	57 (12.8%)	48 (8.1%)	13 (7.6%)	**0.03**
Publications in academic/medical journals	898 (74.4%)	326 (73.1%)	453 (76.8%)	119 (69.6%)	0.12

Trusted for vaccine information:					
News media	102 (8.5%)	47 (10.5%)	51 (8.6%)	4 (2.3%)	**<0.01**
Social media	41 (3.4%)	19 (4.3%)	18 (3.1%)	4 (2.3%)	0.40
Academic/medical journals	1,052 (87.2%)	390 (87.4%)	522 (88.5%)	140 (81.9%)	0.07
Academic/medical institutions	1,029 (85.3%)	386 (86.5%)	509 (86.3%)	134 (78.4%)	**0.02**
Professional medical organizations	1,023 (84.8%)	387 (86.8%)	502 (85.1%)	134 (78.4%)	**0.03**
Other healthcare providers	707 (58.6%)	261 (58.5%)	355 (60.2%)	91 (53.2%)	0.27
Centers for Disease Control and Prevention (CDC)	933 (77.3%)	345 (77.4%)	469 (79.5%)	119 (69.6%)	**0.03**
Food and Drug Administration (FDA)	886 (73.4%)	333 (74.7%)	438 (74.2%)	115 (67.3%)	0.14
State and local public health departments	870 (72.1%)	328 (73.5%)	427 (72.4%)	115 (67.3%)	0.29
Vaccine-focused non-profit organizations	532 (44.1%)	208 (46.6%)	258 (43.7%)	66 (38.6%)	0.19
Often used to share vaccine information with patients:					
One-on-one conversation	1,032 (85.5%)	364 (81.6%)	514 (87.1%)	154 (90.1%)	**<0.01**
Email newsletter	79 (6.5%)	40 (9.0%)	34 (5.8%)	5 (2.9%)	**0.01**
My social media accounts	51 (4.2%)	27 (6.1%)	16 (2.7%)	8 (4.7%)	**0.03**
Other credible social media accounts	57 (4.7%)	31 (7.0%)	20 (3.4%)	6 (3.5%)	**0.02**
My (or my practice’s) website	276 (22.9%)	109 (24.4%)	135 (22.9%)	32 (18.7%)	0.32
Other credible websites	291 (24.1%)	126 (28.3%)	130 (22.0%)	35 (20.5%)	**0.03**
Videos	98 (8.1%)	47 (10.5%)	42 (7.1%)	9 (5.3%)	**0.05**
Posters/flyers/brochures in office	413 (34.2%)	149 (33.4%)	200 (33.9%)	64 (37.4%)	0.63
It's easy to stay up-to-date on vaccine recommendations, contraindications, controversies	812 (67.3%)	290 (65.0%)	414 (70.2%)	108 (63.2%)	0.10
Patients sometimes ask vaccine questions to which you are unsure of the scientific answer	810 (67.1%)	288 (64.6%)	404 (68.5%)	118 (69.0%)	0.35
It'd be helpful to know a patient's vaccine intent and concerns prior to a visit	1,040 (86.2%)	397 (89.0%)	502 (85.1%)	141 (82.5%)	0.06
Feel well prepared for vaccine conversations with patients	1,047 (86.7%)	381 (85.4%)	514 (87.1%)	152 (88.9%)	0.49
Have everything needed to share vaccine info with patients	922 (76.4%)	354 (79.4%)	444 (75.3%)	124 (72.5%)	0.13
More information would help me recommend COVID-19 vaccines to my patients	192 (21.8%)	75 (21.6%)	87 (21.3%)	30 (23.8%)	0.84
Interest in a CME module on how to discuss COVID-19 and other vaccines with patients	472 (39.1%)	153 (34.3%)	243 (41.2%)	76 (44.4%)	0.02
Interest in online resource for HCP detailing how to talk with patients, vaccine recommendations, and vaccine safety issues	792 (65.6%)	283 (63.5%)	405 (68.6%)	104 (60.8%)	0.08
Interest in website to refer patients to that provides them regularly updated and individually tailored vaccine info	798 (66.1%)	283 (63.5%)	407 (69.0%)	108 (63.2%)	0.12
Adverse Event Reporting [c]					
Familiar with VAERS	566 (93.7%)	223 (94.5%)	274 (92.9%)	69 (94.5%)	0.72
Familiar with (fictitious) IARM system	475 (78.8%)	162 (77.1%)	234 (79.3%)	79 (80.6%)	0.75
Ever reported to VAERS	195 (34.5%)	75 (33.6%)	94 (34.3%)	26 (37.7%)	0.82

Ever reported to (fictitious) IARM system	120 (25.3%)	41 (25.3%)	61 (26.1%)	18 (22.8%)	0.84
COVID-19 vaccine recommendations by vaccine					
Pfizer	403 (33.4%)	142 (31.8%)	203 (34.4%)	58 (33.9%)	0.68
Moderna	480 (39.8%)	184 (41.3%)	233 (39.5%)	63 (36.8%)	0.59
Johnson & Johnson	85 (7.0%)	40 (9.0%)	36 (6.1%)	9 (5.3%)	0.13
Novavax	107 (8.9%)	47 (10.5%)	52 (8.8%)	8 (4.7%)	0.07
COVID-19 vaccine recommendations for specific patient populations					
high-risk patients	790 (65.5%)	309 (69.3%)	380 (64.4%)	101 (59.1%)	**0.04**
patients that live with or care for high-risk persons	803 (66.5%)	312 (70.0%)	384 (65.1%)	107 (62.6%)	0.13
6-23-month-old patients	265 (29.0%)	118 (34.9%)	124 (27.6%)	23 (18.4%)	**<0.01**
2-4-year-old patients	311 (32.3%)	144 (39.9%)	135 (29.0%)	32 (23.7%)	**<0.01**
5-11-year-old patients	431 (41.9%)	186 (48.4%)	201 (40.5%)	44 (29.7%)	**<0.01**
12-15-year-old patients	499 (47.3%)	210 (53.7%)	235 (46.0%)	54 (35.1%)	**<0.01**
16-24-year-old patients	607 (51.7%)	250 (57.6%)	289 (50.5%)	68 (40.5%)	**<0.01**
25-64-year-old patients	593 (61.6%)	233 (65.3%)	278 (61.2%)	82 (53.9%)	0.06
65+ year-old patients	748 (78.7%)	286 (81.5%)	358 (79.6%)	104 (69.3%)	**<0.01**
Routine vaccine recommendations for eligible patients by vaccine					
influenza	853 (70.7%)	322 (72.2%)	417 (70.7%)	114 (66.7%)	0.40
routine childhood (e.g., MMR, DTaP)	887 (82.9%)	325 (81.3%)	442 (85.2%)	120 (79.5%)	0.14
HPV	674 (59.3%)	256 (60.4%)	336 (61.0%)	82 (50.9%)	0.06
shingles	644 (66.2%)	254 (69.0%)	295 (65.4%)	95 (61.7%)	0.24
pneumococcal	944 (79.6%)	347 (79.0%)	470 (81.3%)	127 (75.1%)	0.20
Obstacles to administering COVID-19 vaccines:					
believe not needed for some patients	177 (14.7%)	73 (16.4%)	79 (13.4%)	25 (14.6%)	0.41
time it takes to discuss with patients	180 (14.9%)	70 (15.7%)	87 (14.7%)	23 (13.5%)	0.77
uncertainty amount adequate reimbursement	156 (12.9%)	53 (11.9%)	92 (15.6%)	11 (6.4%)	**<0.01**
general administrative burden	370 (30.7%)	127 (28.5%)	195 (33.1%)	48 (28.1%)	0.21
additional workload of another vaccine	325 (26.9%)	117 (26.2%)	171 (29.0%)	37 (21.6%)	0.15
patient concerns about COVID-19 vaccine safety	729 (60.4%)	267 (59.9%)	349 (59.2%)	113 (66.1%)	0.25
patient concerns about general vaccine safety	575 (47.6%)	218 (48.9%)	276 (46.8%)	81 (47.4%)	0.80
patient concerns about COVID-19 vaccine necessity	776 (64.3%)	283 (63.5%)	375 (63.6%)	118 (69.0%)	0.38
patient concerns about COVID-19 vaccine effectiveness	654 (54.2%)	238 (53.4%)	309 (52.4%)	107 (62.6%)	0.06

[a] PA = Physician Assistant; NP = Nurse Practitioner

[b] boldface indicates statistical significance ($p<0.05$) using Pearson's Chi-Squared Test

[c] VAERS = Vaccine Adverse Event Reporting System; IARM = Immunization Adverse Reaction Monitoring (fictitious)

Supplementary Table 8. ***Changes in Survey Responses between September 2021 and January 2023 by Type of Healthcare Personnel***

Survey items	Total		Pediatrician		Family Medicine		PA, NP, Nurse[a]		Pharmacist	
	% Change	P-value[b]	% Change	P-value[b]	% Change	P-value[b]	% Change	P-value[b]	% Change	P-value[b]
High Trust in CDC [c]	-6	**<0.01**	-3	0.38	-5	0.18	-5	0.18	-10	**0.01**
Included telehealth visits before March 2020	3	**0.03**	4	0.19	8	**0.02**	2	0.53	-1	0.75
Included telehealth visits since March 2020	2	0.25	-2	0.34	-3	0.08	6	**0.01**	-1	0.89
Plan to continue telehealth after pandemic	4	**0.01**	4	0.24	1	0.60	5	0.09	6	0.19
Decreased ability to provide routine vaccination due to telehealth	-1	0.61	4	0.37	-9	**0.04**	-5	0.30	7	0.29
Practice currently administers vaccines	1	0.19	2	0.10	2	0.12	0	0.96	0	0.86
Practice participates in the Vaccines for Children (VFC) program	2	0.48	1	0.86	-3	0.55	4	0.34	3	0.44
Since March 2020, practice implemented changes to boost vaccination	-1	0.49	-2	0.60	2	0.63	-4	0.29	2	0.52
patient-focused	0	0.93	-1	0.90	-2	0.74	4	0.44	0	0.93
provider-focused	8	**<0.01**	13	**0.03**	10	0.11	5	0.39	5	0.28
practice-focused	5	0.09	-1	0.90	6	0.34	3	0.56	9	0.06
improved vaccine availability and access	9	**<0.01**	11	0.06	12	0.06	12	**0.04**	5	0.24
Practice stopped routine vaccines since March 2020	-3	0.17	1	0.73	-5	0.28	-2	0.63	-5	0.18
Practice provided seasonal influenza vaccination: 2019-2020	-2	0.14	-1	0.78	-6	0.10	-1	0.69	-2	0.51
Practice provided seasonal influenza vaccination: 2020-2021	1	0.32	1	0.39	4	0.25	-3	0.35	3	0.15
Obstacles to vaccinating during pandemic so far:										
decreased access to patients	-7	**<0.01**	-8	**0.02**	-9	**0.02**	-9	**0.03**	-4	0.28
lenient enforcement of school immunization requirements	5	**0.01**	6	0.12	4	0.29	4	0.32	5	0.16
disruption of vaccine supply	15	**<0.01**	13	**<0.01**	15	**<0.01**	17	**<0.01**	13	**<0.01**
disruption of vaccination due to the staffing and PPE shortages	6	**<0.01**	3	0.46	4	0.29	5	0.19	11	**<0.01**
Obstacles to vaccinating expected in the future:										
decreased access to patients	-17	**<0.01**	-20	**<0.01**	-16	**<0.01**	-16	**<0.01**	-13	**0.01**
lenient enforcement of school immunization requirements	4	0.26	10	0.14	-1	0.92	5	0.54	1	0.91
disruption of vaccine supply	4	0.33	20	**0.03**	6	0.41	-1	0.94	-3	0.61
staffing and PPE shortages	6	0.10	23	**0.01**	4	0.63	8	0.23	-2	0.64

Adverse Event Reporting [d]										
Familiar with VAERS	1	0.55	2	0.15	0	0.96	4	0.33	0	0.97
Familiar with (fictitious) IARM system	6	**0.02**	5	0.28	12	**0.03**	5	0.31	3	0.49
Ever reported to VAERS	-2	0.45	-3	0.65	-2	0.73	3	0.55	-4	0.54
Ever reported to (fictitious) IARM system	0	0.93	5	0.45	-10	0.09	1	0.88	6	0.35
Received at least one COVID-19 vaccine	3	**<0.01**	3	**0.01**	2	0.21	6	**0.01**	2	0.44
Reasons for not vaccinating among HCP not vaccinated/boosted against COVID-19:										
medical condition, temporary	-9	**0.02**	-13	0.13	-3	0.69	-11	**0.04**	-12	0.18
medical condition, permanent	0	0.95	-2	0.89	7	0.54	-6	0.31	5	0.48
concern, side effects	-3	0.61	3	0.91	-20	0.23	-2	0.81	5	0.67
uncomfortable emergency-use authorized vaccine	-6	0.31	5	0.53	-5	0.74	-10	0.27	-5	0.65
vaccine developed/approved too quickly	-6	0.29	-9	0.59	-11	0.49	4	0.67	-17	0.10
distrust due to racism/discrimination/ethics	-4	0.31	5	0.53	-18	0.18	-7	0.27	5	0.48
vaccine trials did not include people like me	-2	0.20	0		8	0.33	-5	**0.04**	-4	0.07
want to wait until more people get vaccine	-19	**<0.01**	-7	0.53	-7	0.60	-22	**<0.01**	-26	**<0.01**
I have low risk for contracting COVID-19	-3	0.66	9	0.62	6	0.71	-5	0.59	-8	0.47
Obstacles to administering COVID-19 vaccines:										
believe not needed for some patients	-12	**<0.01**	-15	0.17	-20	0.06	-15	**0.03**	3	0.71
time it takes to discuss with patients	-6	0.14	15	0.23	-17	0.11	-11	0.06	-1	0.86
uncertainty amount adequate reimbursement	-8	**0.04**	16	0.23	-29	**<0.01**	-15	**<0.01**	2	0.80
general administrative burden	3	0.51	7	0.66	0	1.00	-12	0.11	22	**0.03**
additional workload of another vaccine	-3	0.60	-2	0.89	-22	0.07	-6	0.41	14	0.20
patient concerns about COVID-19 vaccine safety	1	0.84	31	0.06	-18	0.19	-5	0.53	6	0.61
patient concerns about general vaccine safety	-12	**0.04**	-3	0.88	-30	**0.04**	-12	0.15	-2	0.81
patient concerns about COVID-19 vaccine necessity	8	0.17	38	**0.02**	16	0.27	-6	0.47	8	0.47
patient concerns about COVID-19 vaccine effectiveness	-1	0.81	34	0.06	-4	0.80	-7	0.38	-3	0.79
COVID-19 vaccination should be mandated for healthcare workers	-18	**<0.01**	-20	**<0.01**	-18	**<0.01**	-21	**<0.01**	-16	**<0.01**
Regularly taken care of COVID-19 patients	18	**<0.01**	18	**<0.01**	11	**<0.01**	13	**<0.01**	28	**<0.01**
COVID-19 vaccine recommendations for specific patient populations										
high-risk patients	-5	**<0.01**	-3	0.25	-6	**0.04**	-5	0.09	-6	0.06
patients that live with or care for high-risk persons	-7	**<0.01**	-5	0.05	-8	**<0.01**	-5	0.08	-10	**<0.01**
12-15-year-old patients	-7	**<0.01**	-5	0.08	-10	**0.01**	-6	0.21	-10	**0.01**
16-24-year-old patients	-12	**<0.01**	-4	0.09	-14	**<0.01**	-15	**<0.01**	-17	**<0.01**
25-64-year-old patients	-9	**<0.01**	0	0.99	-9	**<0.01**	-8	**0.02**	-11	**<0.01**

65+ year-old patients	-4	**<0.01**	3	0.32	-3	0.08	-4	0.18	-7	**<0.01**
12-15-year-old high-risk patients	-5	**<0.01**	-1	0.72	-4	0.19	-5	0.20	-13	**<0.01**
16-24-year-old high-risk patients	-6	**<0.01**	-1	0.57	-4	0.10	-6	0.08	-12	**<0.01**
25-64-year-old high-risk patients	-5	**<0.01**	2	0.41	-3	0.13	-3	0.28	-9	**<0.01**
65+ year-old high-risk patients	-4	**<0.01**	2	0.37	-3	0.08	-2	0.49	-7	**<0.01**
COVID-19 vaccine recommendations by vaccine										
Pfizer	-31	**<0.01**	-32	**<0.01**	-24	**<0.01**	-35	**<0.01**	-34	**<0.01**
Moderna	-23	**<0.01**	-22	**<0.01**	-19	**<0.01**	-29	**<0.01**	-23	**<0.01**
Johnson & Johnson	-30	**<0.01**	-32	**<0.01**	-35	**<0.01**	-21	**<0.01**	-31	**<0.01**
Routinely co-administering COVID-19 vaccines with other recommended vaccines	0	0.86	4	0.35	5	0.29	-4	0.39	0	0.94
Practice provides COVID-19 vaccines	6	**<0.01**	19	**<0.01**	6	0.19	6	0.11	-3	0.17
Pfizer	9	**<0.01**	1	0.63	10	0.05	13	**<0.01**	10	**<0.01**
Moderna	7	**<0.01**	18	**<0.01**	5	0.40	13	**<0.01**	3	0.42
Johnson & Johnson	-15	**<0.01**	-7	**0.01**	-18	**<0.01**	-4	0.36	-24	**<0.01**
Strategies used to improve COVID-19 vaccine series completion:										
paper-based reminder card	-13	**<0.01**	-11	**0.04**	-13	**0.03**	-15	**<0.01**	-10	**0.02**
reminder telephone calls	-14	**<0.01**	-15	**0.01**	-21	**<0.01**	-9	0.08	-13	**<0.01**
flagging patient charts	-2	0.47	2	0.74	4	0.54	-5	0.27	-7	0.06
scheduling next dose at current visit	-14	**<0.01**	-4	0.29	-19	**<0.01**	-7	0.07	-24	**<0.01**
computerized immunization database/registry	-12	**<0.01**	-4	0.42	-12	0.05	-1	0.86	-24	**<0.01**
More information would help me recommend COVID-19 vaccines to my patients	-4	0.08	-6	0.18	6	0.25	-7	0.11	-5	0.22
Routine vaccine recommendations for eligible patients by vaccine										
influenza	-4	**<0.01**	-3	0.10	-2	0.41	-5	0.05	-7	**0.01**
routine childhood (e.g., MMR, DTaP)	-2	**0.04**	-1	0.44	1	0.66	-4	0.15	-6	0.08
HPV	-5	**0.01**	-3	0.10	-4	0.16	-9	**0.02**	-5	0.26
shingles	-4	**<0.01**	-6	0.20	-3	0.23	-6	**0.04**	-4	0.10
pneumococcal	-1	0.37	0	0.88	1	0.54	-2	0.35	-3	0.20
Interest in a CME module on how to discuss COVID-19 and other vaccines with patients	-7	**<0.01**	-10	0.05	-8	0.15	-7	0.13	-3	0.46
Interest in online resource for HCP detailing how to talk with patients, vaccine recommendations, and vaccine safety issues	19	**<0.01**	23	**<0.01**	25	**<0.01**	14	**<0.01**	16	**<0.01**

[a] PA = Physician Assistant; NP = Nurse Practitioner

[b] boldface indicates statistical significance ($p<0.05$) using Pearson's Chi-Squared Test

[c] CDC = Centers for Disease Control and Prevention; see Supplementary Table 1
[d] VAERS = Vaccine Adverse Event Reporting System; IARM = Immunization Adverse Reaction Monitoring (fictitious)
