# Supplementary material for: Changes in vaccine attitudes and recommendations among US Healthcare Personnel during the COVID-19 pandemic
Source: NPJ Vaccines. 2024 Feb 28;9:49. doi: 10.1038/s41541-024-00826-y (PMC10901873; doi:10.1038/s41541-024-00826-y)
Supplement: Supplementary file 2 — REPORTING SUMMARY [file 41541_2024_826_MOESM2_ESM.pdf]

nature portfolio

Corresponding author(s): Dudley, MZ

Last updated by author(s): November 21, 2023

Reporting Summary

Nature Portfolio wishes to improve the reproducibility of the work that we publish. This form provides structure for consistency and transparency in reporting. For further information on Nature Portfolio policies, see our Editorial Policies and the Editorial Policy Checklist.

Statistics

For all statistical analyses, confirm that the following items are present in the figure legend, table legend, main text, or Methods section.

n/a	Confirmed	
☐	☒	The exact sample size (*n*) for each experimental group/condition, given as a discrete number and unit of measurement
☐	☒	A statement on whether measurements were taken from distinct samples or whether the same sample was measured repeatedly
☐	☒	The statistical test(s) used AND whether they are one- or two-sided<br>*Only common tests should be described solely by name; describe more complex techniques in the Methods section.*
☒	☐	A description of all covariates tested
☐	☒	A description of any assumptions or corrections, such as tests of normality and adjustment for multiple comparisons
☐	☒	A full description of the statistical parameters including central tendency (e.g. means) or other basic estimates (e.g. regression coefficient) AND variation (e.g. standard deviation) or associated estimates of uncertainty (e.g. confidence intervals)
☒	☐	For null hypothesis testing, the test statistic (e.g. *F*, *t*, *r*) with confidence intervals, effect sizes, degrees of freedom and *P* value noted<br>*Give P values as exact values whenever suitable.*
☒	☐	For Bayesian analysis, information on the choice of priors and Markov chain Monte Carlo settings
☒	☐	For hierarchical and complex designs, identification of the appropriate level for tests and full reporting of outcomes
☐	☒	Estimates of effect sizes (e.g. Cohen's *d*, Pearson's *r*), indicating how they were calculated

Our web collection on statistics for biologists contains articles on many of the points above.

Software and code

Policy information about availability of computer code

Data collection	*Provide a description of all commercial, open source and custom code used to collect the data in this study, specifying the version used OR state that no software was used.*
Data analysis	Stata version 16

For manuscripts utilizing custom algorithms or software that are central to the research but not yet described in published literature, software must be made available to editors and reviewers. We strongly encourage code deposition in a community repository (e.g. GitHub). See the Nature Portfolio guidelines for submitting code & software for further information.

Data

Policy information about availability of data

All manuscripts must include a data availability statement. This statement should provide the following information, where applicable:

- Accession codes, unique identifiers, or web links for publicly available datasets
- A description of any restrictions on data availability
- For clinical datasets or third party data, please ensure that the statement adheres to our policy

Deidentified individual participant data will not be made available.

Research involving human participants, their data, or biological material

Policy information about studies with human participants or human data. See also policy information about sex, gender (identity/presentation), and sexual orientation and race, ethnicity and racism.

Reporting on sex and gender	*Use the terms sex (biological attribute) and gender (shaped by social and cultural circumstances) carefully in order to avoid confusing both terms. Indicate if findings apply to only one sex or gender; describe whether sex and gender were considered in study design; whether sex and/or gender was determined based on self-reporting or assigned and methods used. Provide in the source data disaggregated sex and gender data, where this information has been collected, and if consent has been obtained for sharing of individual-level data; provide overall numbers in this Reporting Summary. Please state if this information has not been collected. Report sex- and gender-based analyses where performed, justify reasons for lack of sex- and gender-based analysis.*
Reporting on race, ethnicity, or other socially relevant groupings	We calculated both crude and adjusted ORs and CIs, to control for potential confounding of sociodemographic characteristics shown to be associated with COVID-19 booster status in this analysis (Appendix 2) and previously,4 such as graduation year (to approximate age), race/ethnicity, region, and practice location (urban/suburban/rural). Given the small numbers in each strata, we took a conservative approach and ran 100 bootstrap replications to calculate standard errors.
Population characteristics	we recruited family medicine doctors, pediatricians, pharmacists, physician assistants (PAs), nurse practitioners (NPs), and nurses (including registered nurses and licensed practical nurses) who provided care directly to patients,
Recruitment	From December 20, 2022, to January 25, 2023, we recruited family medicine doctors, pediatricians, pharmacists, physician assistants (PAs), nurse practitioners (NPs), and nurses (including registered nurses and licensed practical nurses) who provided care directly to patients, using SurveyHealthcareGlobus.25 SurveyHealthcareGlobus, a double opt-in network panel, comes from a population of more than 2 million people and is regularly validated and updated from standard core sources including medical and hospital directories, verified healthcare websites, and the American Medical Association. Recruitment only ceased after at least 300 HCP of each of the four main categories (pediatricians, family medicine doctors, PAs/NPs/ nurses, and pharmacists) were surveyed. Respondents were given a $10 USD honorarium. Participants were members of the SurveyHealthcareGlobus panel and gave their consent to be surveyed.
Ethics oversight	The Johns Hopkins Bloomberg School of Public Health Institutional Review Board considered this study public health surveillance and not human subject research.

Note that full information on the approval of the study protocol must also be provided in the manuscript.

Field-specific reporting

Please select the one below that is the best fit for your research. If you are not sure, read the appropriate sections before making your selection.

☐ Life sciences ☒ Behavioural & social sciences ☐ Ecological, evolutionary & environmental sciences

For a reference copy of the document with all sections, see nature.com/documents/nr-reporting-summary-flat.pdf

Behavioural & social sciences study design

All studies must disclose on these points even when the disclosure is negative.

Study description	cross-sectional survey of HCP conducted in January 2023, which was more than a year since COVID-19 booster doses were first authorized
Research sample	family medicine doctors, pediatricians, pharmacists, physician assistants (PAs), nurse practitioners (NPs), and nurses (including registered nurses and licensed practical nurses) who provided care directly to patients
Sampling strategy	Recruitment only ceased after at least 300 HCP of each of the four main categories (pediatricians, family medicine doctors, PAs/NPs/ nurses, and pharmacists) were surveyed
Data collection	SurveyHealthcareGlobus, a double opt-in network panel, comes from a population of more than 2 million people and is regularly validated and updated from standard core sources including medical and hospital directories, verified healthcare websites, and the American Medical Association
Timing	December 20, 2022, to January 25, 2023
Data exclusions	NA
Non-participation	NA
Randomization	NA

Reporting for specific materials, systems and methods

We require information from authors about some types of materials, experimental systems and methods used in many studies. Here, indicate whether each material, system or method listed is relevant to your study. If you are not sure if a list item applies to your research, read the appropriate section before selecting a response.

Materials & experimental systems

n/a	Involved in the study
☒	☐ Antibodies
☒	☐ Eukaryotic cell lines
☒	☐ Palaeontology and archaeology
☒	☐ Animals and other organisms
☒	☐ Clinical data
☒	☐ Dual use research of concern
☒	☐ Plants

Methods

n/a	Involved in the study
☒	☐ ChIP-seq
☒	☐ Flow cytometry
☒	☐ MRI-based neuroimaging

Plants

Seed stocks	*Report on the source of all seed stocks or other plant material used. If applicable, state the seed stock centre and catalogue number. If plant specimens were collected from the field, describe the collection location, date and sampling procedures.*
Novel plant genotypes	*Describe the methods by which all novel plant genotypes were produced. This includes those generated by transgenic approaches, gene editing, chemical/radiation-based mutagenesis and hybridization. For transgenic lines, describe the transformation method, the number of independent lines analyzed and the generation upon which experiments were performed. For gene-edited lines, describe the editor used, the endogenous sequence targeted for editing, the targeting guide RNA sequence (if applicable) and how the editor was applied.*
Authentication	*Describe any authentication procedures for each seed stock used or novel genotype generated. Describe any experiments used to assess the effect of a mutation and, where applicable, how potential secondary effects (e.g. second site T-DNA insertions, mosiacism, off-target gene editing) were examined.*
